# Subcortical segmentation of the fetal brain in 3D ultrasound using deep learning

**DOI:** 10.1016/j.neuroimage.2022.119117

**Published:** 2022-03-21

**Authors:** Linde S. Hesse, Moska Aliasi, Felipe Moser, the INTERGROWTH-21st Consortium, Monique C. Haak, Weidi Xie, Mark Jenkinson, Ana I.L. Namburete

**Affiliations:** a Institute of Biomedical Engineering, Department of Engineering Science, University of Oxford, United Kingdom; b Department of Obstetrics and Fetal Medicine, Leiden University Medical Center, The Netherlands; c Visual Geometry Group, Department of Engineering Science, University of Oxford, United Kingdom; d Wellcome center for Integrative NeuroImaging, FMRIB, University of Oxford, United Kingdom; e Australian Institute for Machine Learning (AIML), Australia; f South Australian Health and Medical Research Institute (SAHMRI), Australia; g Department of Computer Science, University of Oxford, United Kingdom

**Keywords:** Ultrasound, Segmentation, Fetal brain, Subcortical, Few-Shot learning, Deep learning

## Abstract

The quantification of subcortical volume development from 3D fetal ultrasound can provide important diagnostic information during pregnancy monitoring. However, manual segmentation of subcortical structures in ultrasound volumes is time-consuming and challenging due to low soft tissue contrast, speckle and shadowing artifacts. For this reason, we developed a convolutional neural network (CNN) for the automated segmentation of the choroid plexus (CP), lateral posterior ventricle horns (LPVH), cavum septum pellucidum et vergae (CSPV), and cerebellum (CB) from 3D ultrasound. As ground-truth labels are scarce and expensive to obtain, we applied few-shot learning, in which only a small number of manual annotations (n = 9) are used to train a CNN. We compared training a CNN with only a few individually annotated volumes versus many weakly labelled volumes obtained from atlas-based segmentations. This showed that segmentation performance close to intra-observer variability can be obtained with only a handful of manual annotations. Finally, the trained models were applied to a large number (n = 278) of ultrasound image volumes of a diverse, healthy population, obtaining novel US-specific growth curves of the respective structures during the second trimester of gestation.

## 1 Introduction

During pregnancy, several subcortical structures in the fetal brain are assessed with ultrasound (US) imaging. Especially earlier in gestation, when the fetal skull is not yet fully calcified, the US beam can penetrate the skull and visualize the subcortical structures. The abnormal development of subcortical structures can be a potential sign of a severe neurological condition and, as such, it is important to monitor their development during gestation. Brain development can be studied in detail with targeted fetal neurosonography (Paladini et al., 2021), however, this is only performed in fetuses at high-risk for CNS abnormalities and is not part of routine obstetric examinations. For this reason, efforts should be made to develop analysis methods that can improve subcortical assessment during routine pregnancy monitoring.

Subcortical structure development is best quantitatively assessed using 3D volumetric information. However, to date, most studies analyzing these structures during gestation only use in-plane measurements obtained from 2D US (Shinohara et al., 2020; Tongsong et al., 1999). These measurements are routinely acquired during a basic examination of the fetal brain (Malinger et al., 2020) but only provide a limited representation of the anatomical development and morphology. For this reason, it is desirable to use 3D US to obtain volumetric measures of subcortical development. As manually annotating several structures is not feasible in routine clinical practice, automated subcortical segmentation methods for 3D US could facilitate this analysis and may provide new insights into in utero subcortical development.

Structural segmentation in fetal brain US is a challenging task due to low soft tissue contrast, reverberation artifacts and the characteristic presence of speckle. Consequently, precise structural boundaries can be hard to distinguish, resulting in high inter- and intra-observer variability in manual annotations. Since manual segmentation is not a task usually performed in clinical practice, even trained ultrasonographers can have difficulty in accurately segmenting subcortical structures in 3D US volumes. An additional challenge of US volumes acquired with a free-hand scanning protocol, as is typical at the bedside, is the varying position of the fetal brain due to the unpredictable fetal position in the womb as well as movement of the transducer relative to the fetal head. Furthermore, due to interactions of the US beam with the fetal skull, typically only the cerebral hemisphere distal to the US transducer is well visible in the US volumes.

Recently, it has been shown that deep learning methods can be successfully applied to other segmentation tasks in 3D US volumes of the fetal brain (Hesse and Namburete, 2020; Moser et al., 2019; Venturini et al., 2020; Wyburd et al., 2020), resulting in higher performance than traditional image analysis methods. However, due to the difficulty of obtaining manual annotations for subcortical structures, a key barrier to applying deep learning methods to this task is obtaining sufficient ground-truth annotations for training. Therefore, to the best of our knowledge, only very few previous studies have applied deep learning to subcortical structure segmentation in 3D US, which will be discussed in more detail in Section 2.1.

To overcome the need for a large manually annotated dataset, few-shot learning can be used, in which only a small number of manual annotations are used to train a convolutional neural network (CNN). Several few-shot learning approaches have been proposed for segmentation tasks in the medical image domain (Al Chanti et al., 2021; Mondal et al., 2018; Roy et al., 2020), showing that good segmentation performance can be obtained using only a very limited amount of voxel-wise manual annotation. In this work, we will use few-shot learning to develop a deep-learning based method for the segmentation of several subcortical structures in 3D US. To the best of our knowledge, few-shot learning has not been applied for this task, and, as such, this will be the first study exploring subcortical segmentation of the fetal brain in a low-data regime. Specifically, we will compare two types of ground-truth labels for training, where both have been obtained using only a few manual annotations: (1) a small number of individually annotated US volumes (referred to as *expert labels*), and (2) a large number of weakly labelled US volumes obtained by propagating annotated template images (referred to as *atlas labels*).

Additionally, we investigate the impact of image alignment to a common coordinate system as a pre-processing step. As the anatomical orientation of the fetal brain varies in US image volumes, initial global (affine) registration of the brain is expected to have a positive effect on the segmentation performance. Although the alignment of US volumes is a non-trivial task, methods have been developed for the automated alignment of fetal brain volumes (Cuingnet et al., 2013; Namburete et al., 2018b). Furthermore, compared to the voxel-wise annotation of several subcortical structures, manual image alignment requires considerably less effort. For these reasons, alignment of the volumes to a common coordinate system can be preferred over the additional voxel-wise manual annotation, and, as such, it is important to explore global alignment as a requisite preprocessing step.

Using few-shot learning, we aim to segment the choroid plexus (CP), lateral posterior ventricle horn (LPVH), cerebellum (CB) and cavum septum pellucidum et vergae (CSPV) during the second trimester of gestation. This trimester is of particular interest since this is when women undergo an US examination as part of routine care to screen for anomalies (Health, 2008). Furthermore, in this trimester most subcortical structures have developed enough to be visible on US but are less affected by acoustic shading due to progressing calcification of the skull and amniotic fluid reduction later in gestation.

Lastly, to obtain an improved understanding of volumetric subcortical development during the second trimester, we will apply our developed segmentation models to a large cohort of healthy fetuses. The resulting model predictions will be validated for the CB, as volumetric growth curves for the CB have been reported in previous studies (Andescavage et al., 2017; Babucci et al., 2019; Chang et al., 2000; Hata et al., 2007; Hatab et al., 2008; Kyriakopoulou et al., 2017; Scott et al., 2012), and subsequently used to generate novel US-specific growth curves of subcortical volume development during the second trimester of gestation.

### 1.1 Clinical subcortical structure assessment

The structures segmented in this study were selected based on their importance during the routine anomaly scan in the second trimester. During this scan, measurements of both the CB and LPVH are performed by the sonographer. The measurement of the CB is referred to as the transverse cerebellar diameter (TCD), and is used to screen for central nervous system (CNS) abnormalities as well as to date the pregnancy (Malinger et al., 2020). The atrial width, which is measured from the LPVH, can indicate ventriculomegaly and is considered to be integral to diagnosing CNS abnormalities (Malinger et al., 2020). Furthermore, the total ventricular volume, of which the LPVH is part, has been shown to be enlarged in fetuses with certain congenital conditions, such as types of congenital heart disease (Khalil et al., 2014). For a similar reason the CP volume is of interest, as this can be used to compute the ventricular cerebrospinal fluid content by subtracting it from the total ventricular volume. Measurements of the CSPV are not routinely performed during an US examination, however, the CSP, which is part of the CSPV, should be visible after 17-20 gestational weeks (GWs) and its absence or irregular shape may indicate a partial agenesis of the corpus callosum (Karl et al., 2017; Malinger et al., 2020; Shen et al., 2015). Furthermore, recent work also suggests that the CSP diameter is larger in small-for-gestational age (SGA) fetuses than in healthy controls (Jacob et al., 2020).

## 2 Related work

Automated subcortical structure segmentation has been widely studied in MRI volumes of the adult brain (Akkus et al., 2017; Dolz et al., 2018; Fischl 2012). However, the adult brain is structurally different to the actively developing fetal brain during gestation, which contains structures in transient stages of morphogenesis. For example, certain structures, such as the CSPV (which is in truth a fluid-filled cavity), are only present during gestation and typically disappear after birth (Dremmen et al., 2019). As MRI can also be safely used to visualize the fetal brain, but is clinically only recommended to confirm or complement a diagnosis following an US examination (Paladini et al., 2021), some studies have performed subcortical structure segmentation in fetal MRI volumes (Gholipour et al., 2017; Khalili et al., 2019; Makropoulos et al., 2018; Payette et al., 2021; Sanroma et al., 2018). Notably, (Gholipour et al., 2017) published an annotated atlas of the fetal brain from 21 to 39 weeks of gestation and applied these labels to perform multi-atlas segmentation for new subjects. However, segmentation methods developed for MRI cannot be directly applied to US volumes due to the very different nature of the image acquisitions. While US is based on the interaction of the sound waves with tissue boundaries, resulting in discontinuous boundaries in the resulting image, MRI is based on protons in the tissue that react to magnetic fields. As a result, intensities on an MRI scan are rather uniform, whereas the intensities on an US scan heavily depend on the distance of the boundary to the probe as well as on the boundaries the US beam has already passed through. For example, the two lateral ventricles will appear very similar on a fetal brain MRI whereas on US typically only one lateral ventricle is well visible due to shadowing of the fetal skull in the proximal hemisphere. As a result of these acquisition differences, there is no direct one-to-one intensity mapping between US and MRI images, which makes it challenging to use fetal MRI atlas labels for US images.

In the remainder of this section we will outline previous work performed for subcortical structure segmentation in US. Furthermore, we will provide a brief overview of studies analyzing volumetric subcortical structure development during gestation.

### 2.1 Automated subcortical segmentation in US

To the best of our knowledge, the first automated subcortical segmentation method for fetal brain US was developed by (Gutiérrez-Becker et al., 2013). In that study, a statistical shape model was used to segment the CB in 3D fetal US volumes. The performance was promising, but it is not clear how this method can be easily extended to other subcortical structures with more shape variation than the CB, making it challenging to develop an initial shape model.

In another study (Yaqub et al., 2013), segmentation of the CP, LPVH, the CSP and the CB was performed in 3D US scans between 18 and 26 weeks of gestation. The authors proposed using a random decision forest using both appearance and distance features. Reported performance was good for the CP, LPVH and CSPV, but their method failed to generalize well to the CB. Furthermore, the search region for each structure was limited to the smallest cuboid enclosing all manual ground-truth annotations from the respective structure. More recently, (Huang et al., 2018) proposed using a region-based based descriptor to segment the CSPV (referred to as corpus callosum in the paper) and CP in 2D US. In contrast to the aforementioned studies, a 2D slice of the full fetal brain was used as input for the segmentation model. However, the sampled 2D slices were limited to standard US planes, making the segmentation substantially less challenging than full 3D segmentation.

Since the introduction of deep learning for image segmentation, most specifically the U-Net architecture (Ronneberger et al., 2015), many segmentation tasks in medical imaging have shown an increase in performance. However, only a few studies have applied deep learning methods to subcortical structure segmentation in fetal US, as usually a relatively large dataset with ground-truth labels is needed. In (Venturini et al., 2019) MRI atlas labels were used to overcome this need for manual annotations. Labels from the respective GW template of the fetal brain MR atlas (Gholipour et al., 2017) were registered to the individual US volumes, resulting in weak labels for the white matter, brainstem, thalami, and CB. These labels were subsequently used to train a multi-label 3D CNN to segment the respective subcortical structures in 3D US volumes. The reported performance was good, however, a severe downside of this study was that testing was performed on the same weak MR atlas labels as used in training. As the registered MR atlas labels can be erroneous, and do not always align well with the structures visible in the US volumes, this therefore yields a biased assessment of performance and as such does not analyze the effect of using weak labels. In (Hesse and Namburete, 2020), active contours were applied to improve atlas-based labels as a pre-processing step before training a 3D U-Net, showing increased performance for CSPV segmentation. However, that method was only validated for an age range between 20 and 24 GWs, and does not easily translate to all subcortical structures. Another method for segmenting the CSPV was presented by (Wu et al., 2020) and used a modified U-Net to perform segmentation in 2D US. However, as in (Huang et al., 2018), segmentation was limited to 2D standard planes. Lastly, in (Venturini et al., 2020) the CB was segmented using unlabeled images in addition to fully annotated images to train a CNN. While best performance was close to intra-observer variability, the number of labeled images required to obtain this performance (n=80) was relatively high. Furthermore, this approach was not extended to any other fetal subcortical structures.

In addition to the previously mentioned methods, some clinical studies report using multiplanar segmentation or Virtual Organ Computer-Aided AnaLysis (VOCAL) software (GE Healthcare) to perform their volume measurements in 3D US (Babucci et al., 2019; Benavides-Serralde et al., 2009; Sotiriadis et al., 2012; Zeng et al., 2015). These methods estimate the structural volume from 2D contours drawn on parallel (multiplanar) or oblique (VOCAL) planes. Although this does speed up analysis compared with fully annotating the volume, it still requires substantial manual annotation and is, as such, not considered to be an automated method.

### 2.2 Subcortical structure development

During a standard fetal US examination, multiple in-plane measurements, such as the TCD, are performed to assess the development and quantify the normality of the brain. For this reason, most studies analyzing subcortical growth during gestation use these 2D measurements (Malinger et al., 2020). Studies that do analyze volumetric development of subcortical structures during gestation most frequently use MRI volumes, either by manually annotating the volumes (Andescavage et al., 2017; Hatab et al., 2008; Scott et al., 2012) or by using an atlas-based approach (Scott et al., 2011). Theoretically, volumetric measures should be independent of the acquisition type (MRI or US), but in practice these measures can vary due to the different appearance of tissues in each modality.

A small number of studies have analyzed 3D subcortical development using US (Babucci et al., 2019; Benavides-Serralde et al., 2009; Chang et al., 2000; Hata et al., 2007; Júnior et al., 2007; Rutten et al., 2009; Sotiriadis et al., 2012; Zeng et al., 2015), and all have used the previously described multiplanar or VOCAL software. In both (Benavides-Serralde et al., 2009 and Zeng et al., 2015), total intracranial, frontal, thalamic and cerebellar volumes were computed to estimate the average volume differences for growth-restricted fetuses (Benavides-Serralde et al., 2009) and fetuses with congenital heart disease (Zeng et al., 2015) compared against a normal control group. Several other studies measured thalamic (Babucci et al., 2019; Sotiriadis et al., 2012) and cerebellar volumes (Babucci et al., 2019; Chang et al., 2000; Hata et al., 2007; Júnior et al., 2007; Rutten et al., 2009) in order to construct normal ranges for subcortical development during gestation.

In summary, even though several studies have proposed subcortical segmentation methods for fetal US, a single method obtaining good performance across multiple structures is lacking for 3D US. Furthermore, due to the absence of accurate automated segmentation methods, only thalamic and cerebellar volumetric development during gestation have been studied in previous work. In this study, we address these limitations by developing a segmentation method aiming to obtain competitive segmentation performance across multiple subcortical structures (CB, LPVH, CSPV and CP) and subsequently applying these methods to obtain novel volumetric growth curves for the respective structures during gestation.

## 3 Methods

In this study, we aim to accurately segment the CB, LPVH, CSPV and CP in 3D US image volumes during the second trimester of gestation using a minimal number of voxel-wise annotations, i.e. few-shot segmentation. Defining *n*_*a*_ as the number of images that is manually annotated, we will consider two types of training labels, both obtained from an equivalent number of manual annotation: (1) training a model naively with *n*_*a*_ individually annotated 3D images, and (2) training a model with a large number of weak propagated atlas labels obtained from annotating *n*_*a*_ 3D template images. These label types are referred to as *expert* and *atlas* labels respectively, and will be abbreviated as *exp* and *atl* in mathematical notation.

### 3.1 Notation

Given a dataset of *m* image volumes, the set of image volumes in their original acquired orientation is denoted as $\overset{˘}{X}=\left\{{\overset{˘}{X}}_{1},\ldots ,{\overset{˘}{X}}_{m}\right\}$ with $\overset{˘}{X}\in {\mathbb{R}}^{3}$, and the set of image volumes rigidly aligned to the same reference coordinate system as $\dot{X}=\left\{{\dot{X}}_{1},\ldots ,{\dot{X}}_{m}\right\}$. Referring to either $\overset{˘}{X}\;$ or $\dot{X}$ will simply be done by using *𝒳* and the original and manually aligned images will also be referred to as *unaligned* and *aligned*, respectively. The ground-truth multi-label segmentation masks are denoted by *Y*. The binary mask of a single class will be denoted by a subscript *c*, with *c* being the class (structure), and the type of the ground-truth label (*atlas* or *expert*) is marked with a superscript $\left(Y_{c}^{type\;}\right)$. A full overview of all notation used in this work can be found in Appendix A.

To avoid ambiguity between *image volumes* and a *volume measurement*, in the remainder of this paper we will refer to our 3D image volumes as *images*.

### 3.2 CNN Architecture

We propose a multi-label 3D U-Net (Ronneberger et al., 2015) with batch normalization (Ioffe and Szegedy, 2015) for the subcortical segmentation. The network, defined by *θ*, predicts a multi-channel segmentation, denoted by *Ŷ*, for a 3D US input image: *Ŷ* = *θ*(*X*), with each channel corresponding to a class. Following the same convention as for the images, $\overset{˘}{\theta }$ and $\dot{\theta }$ correspond to networks trained with $\overset{˘}{X}$ and $\dot{X}$ respectively. To obtain probabilities for each individual class, a soft-max function is applied across the channel dimension of *Ŷ*. Final predictions for a single class, denoted by *Ŷ_c_*, are subsequently obtained by assigning each voxel to the class with the highest probability. Based on initial experiments, we designed our network with a depth of five, and 16 feature maps in the initial block of the encoder. More details on the network architecture can be found in Fig B.2 (Appendix B). However, although we obtained best performance with this network configuration during initial experiments, we are in this study not particularly interested in the effect of the network architecture on the segmentation task but aim to explore the optimal way of training a CNN with a small number of manual annotations.

### 3.3 Few-Shot training labels

#### 3.3.1 Atlas labels

**Fig. 2:**
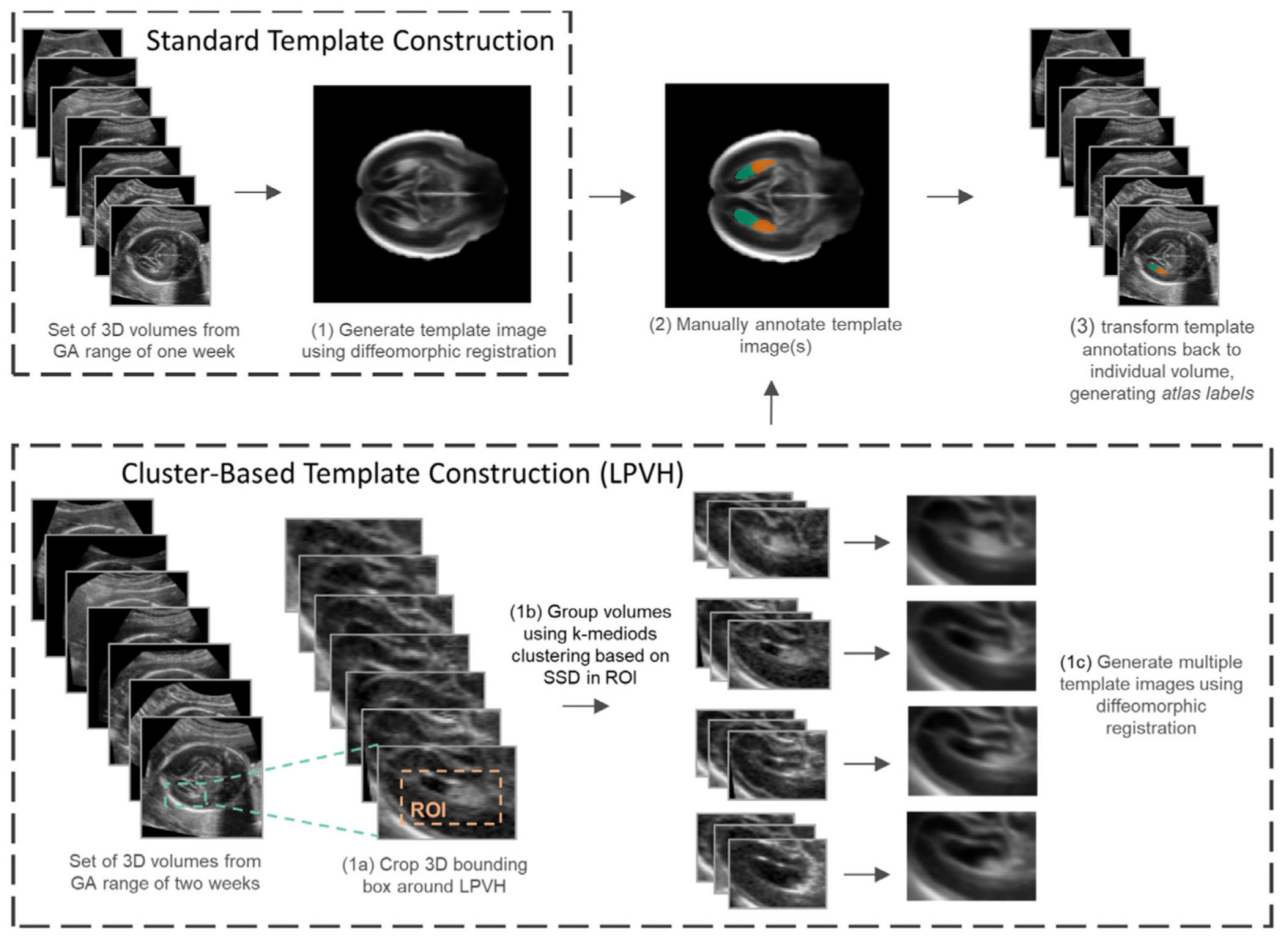
Schematic overview of atlas label generation. Standard whole brain templates (top row) were constructed for each GW and all structures (CB, CP, CSPV and LPVH) were annotated in these templates. Cluster-based template construction (bottom row) was only performed for the LPVH.

Atlas-based labels were obtained for the images by manually annotating 3D template images as opposed to annotating individual images (Fig. 2). Template images were created by registering a subset of *s* rigidly aligned images $\dot{S}\in \dot{X}$ to each other using a Demons diffeomorphic groupwise registration approach (Namburete et al., 2018a), resulting in a set of non-rigid transformations that can transform each image from the rigidly aligned orientation to a reference space *R* defined by: $T=\left\{T_{i}:{\dot{X}}_{i}\to X_{i,R}|i=1,\ldots ,s\right\}$. The registered images were subsequently averaged to obtain a template image $K=\frac{1}{s}\sum_{i=1}^{i=s}X_{i,R}$.

In this template *K*, the four subcortical structures of interest were manually annotated (see Section 3.4), resulting in a set of labelled template images. For simplicity, the annotated templates images will also be referred to with *K*. Subsequently, these labels were non-linearly registered back to the individual images using the inverse transform of the registration to the reference space, given by: $T_{inv}=\{T_{i}^{-1}:K\to {\dot{Y}}_{i}|i=1,\ldots ,s\}$. Due to anatomical variations between individuals and potential errors in the registration, the resulting labels may contain imperfections and are thus considered to be weak labels. To clearly differentiate between the annotated template images (which could be referred to as an atlas) and the propagated atlas-based labels, we will refer to the former only as *annotated template images* and to the latter as *atlas labels*.

#### Standard Template Construction

In order to generate the template images, rigidly aligned images from a GA range of 1 week were used, resulting in one template image per GW. To capture possible differences in anatomy between the left and right cerebral hemispheres, template images for each hemisphere were constructed separately using the respective images and fused together at the midsagittal plane by joining the interhemispheric fissure in the two templates (Fig. 2 top row). The set of annotated template images (one per GW) is denoted by *𝒦*, containing annotations of all segmented structures (LPVH, CB, CP and CSPV).

#### Cluster-Based Template Construction

During initial experiments with the atlas labels, it was seen that the LPVH shape was not well captured by a single template image per GW. For this reason, our method was extended to generate multiple templates for this structure using a clustering approach (Fig. 2 bottom row). We selected images from an age range of two GWs, grouped them using k-mediods clustering and subsequently constructed *n*_*c*_ template images of the LPVH using this grouping. In our experiments *n*_*c*_ was set to four as for this number the resulting template images captured the most important shape variations (based on a visual assessment of the templates). As any anatomical variation between left and right can be captured by the clustering, images of the left and right hemisphere were combined by flipping the right side images across the midline. In the resulting clustered template images (denoted by *K ^clust^*) only the LPVH was manually annotated, resulting in an additional set of atlas labels for the LPVH. Unless explicitly mentioned otherwise, the labels propagated from the cluster-based template images are used for the LPVH in the remainder of this study (i.e. $Y_{LPV\;H}^{atl\;}$ is obtained from the cluster-based template annotation).

To perform the k-mediods clustering, all images were cropped to an area around the LPVH (same bounding box dimensions across the whole GA range) and the intensities of the cropped images were normalized using histogram equalization. The size of this crop was empirically set, and was predominantly performed to reduce computation time. Next, all bounding boxes were pairwise registered to each other (using the same Demons registration used for template construction) and the sum-of-squared-differences (SSD) was computed over a region of interest (ROI) around the LPVH. This ROI was manually set for every two-week gestational window. The resulting square matrix of pairwise SSD values was subsequently used as input for the k-mediods clustering. As k-mediods clustering can be sensitive to outliers, we excluded a few outliers based on their visual appearance prior to clustering. After the clustering step, each outlier was assigned to the nearest cluster based on the distance to the cluster center. Once all images were assigned to a cluster, templates were created from the images in each cluster, including the outliers, as described earlier. The k-mediods clustering was set to repeat 500 times, and results containing clusters with less than three images were discarded.

### 3.4 Manual annotation process

Both the constructed template images, *𝒦* for all structures and *𝒦^clust^* for the LPVH, as well as a subset of individual images, $\dot{X}$, had to be manually annotated. Since subcortical segmentation is not a task usually performed clinically, and structural boundaries can be very challenging to distinguish in 3D US images, a bespoke segmentation protocol was defined in consultation with experienced ultrasonographers (M. Aliasi and M.C. Haak). An overview of the manual segmentation protocol is shown in Table 1. Based on this protocol, the manual segmentations (for both template and individual images) were performed by L.S. Hesse and verified by M. Aliasi. To illustrate the challenging nature of subcortical structural segmentation, an example annotation of the CB across the three orthogonal views can be found in Appendix B (Fig. B.1).

As described before, typically only one hemisphere is well visible in the 3D US images. For this reason, annotation of the CP and LPVH in $\dot{X}$ was only performed in the most visible hemisphere. However, as the whole brain template images (*𝒦*) were created for both the left and right hemisphere separately and subsequently fused together, manual annotation of these images involved annotation of the CP and LPVH in both hemispheres, thus resulting in two manual template annotations per GW for these structures. As both the CSPV and CB are structures near the midsagittal plane, these are less affected by acoustic shadowing and are segmented in full. As a result, when setting an equal *n*_*a*_, defined as the number of manually annotated (template) images, between expert and atlas labels, this results in an equivalent number of annotations for the CB and CSPV, but twice as much annotation of the CP and LPVH for the atlas labels.

The annotation of the LPVH in both left and right hemisphere in the standard template images also means that the annotation effort required to annotate the LPVH in *𝒦*^*clust*^ (four templates per two GWs) was the same as annotating the LPVH in *𝒦* (one template per GW, annotating both the left and right LPVH). For consistency among the structures, for the atlas labels *n*_*a*_ will refer to the number of standard template images that were annotated, which is thus equal to one per GW.

All manual annotations were performed on the aligned images, $\dot{X}$. Labels for $\overset{˘}{X}$ were generated by transforming the labels back to the un-aligned space using the inverse of the rigid manual alignment transform.

### 3.5 Experimental set-Up

#### 3.5.1 Dataset and preprocessing

**Fig. 3:**
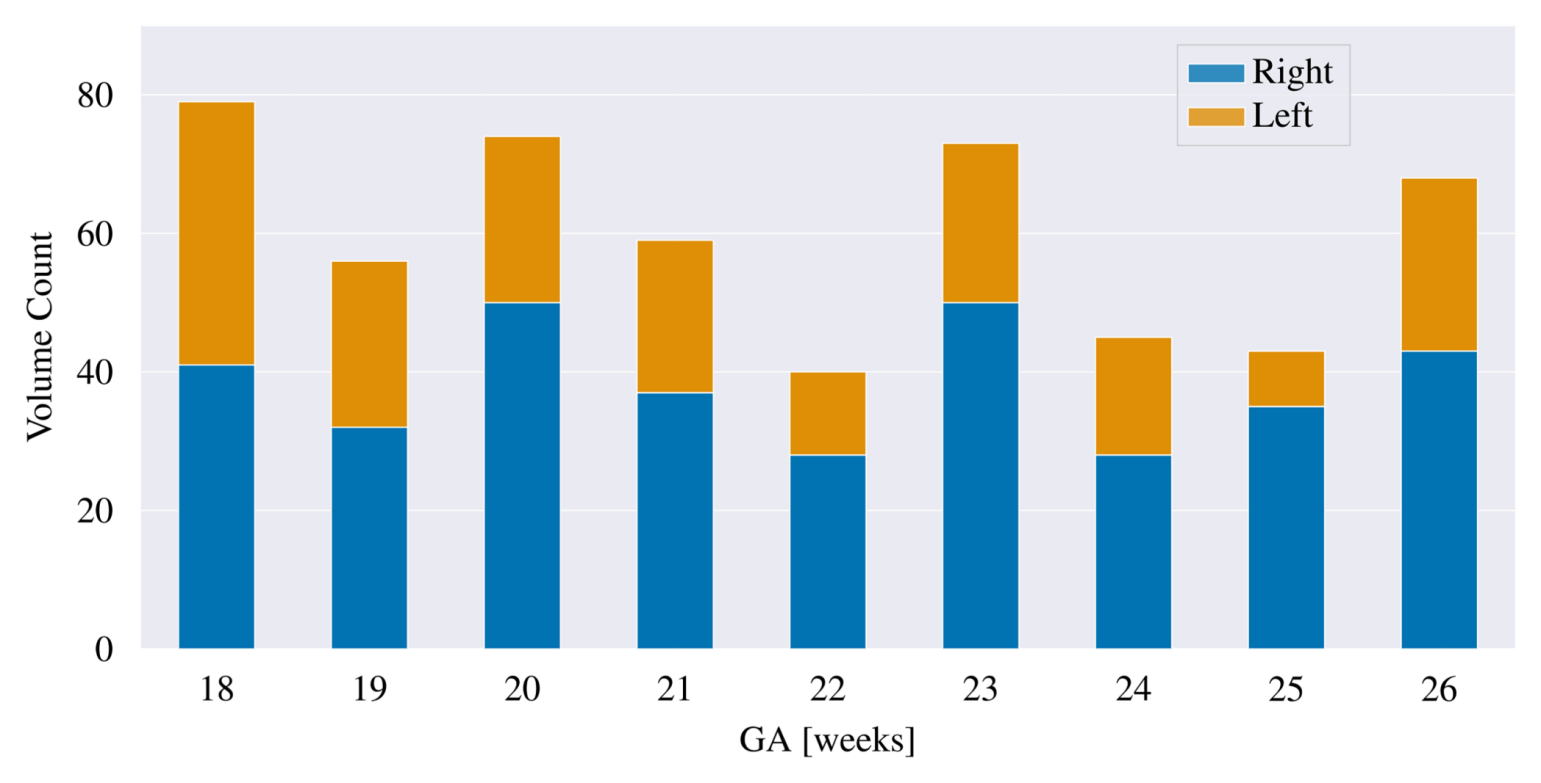
Data distribution of the 537 US images used in this study.

For this study, 3D US images acquired by the INTERGROWTH-21st Fetal Growth Longitudinal Study were used (Papageorghiou et al., 2014). The studys main aim was to describe human growth and neurodevelopment from early pregnancy to 2 years of age in an optimally healthy population drawn from eight urban areas worldwide, geographically delimited to ensure the study was population-based. The large cohort of healthy pregnant women were enrolled before 14 weeks of gestation. All images were acquired from the axial plane on a Philips US machine (Philips HD-9, Philips Ultrasound, USA) with a curvilinear abdominal transducer. We used a total of 537 fetal images acquired between 18 and 26 weeks of gestation and all these fetuses were born without congenital malformations. This age range in the second trimester was selected as the subcortical structures of interest are too small to accurately segment before 18 GWs, whereas after 26 GWs the images in our dataset were heavily affected by reduced amniotic fluid and acoustic shadowing of the fetal skull that becomes increasingly calcified. Furthermore, we ensured that images from both the left and right hemisphere were included and that only images with sufficient US quality were included in this total. In Fig. 3 the data distribution over the GA range is shown.

As an initial pre-processing step, all images were resampled to an isotropic voxel size of 0.6 mm (using trilinear interpolation) and cropped to the same dimensions of 160 × 160 × 160 voxels. Furthermore, all images were manually aligned using a rigid transformation to the same reference space, as the anatomical orientation of the brain varies in the original scan due to transducer and fetal position during acquisition.

The complete dataset consisted of 537 images, of which 259 were used for model development and the remaining 278 to generate volumetric growth curves of the four subcortical structures (referred to as the *analysis* subset or *𝒳*_*analysis*_). Of the 259 images used for model development, 20 were used for testing (*𝒳*_*test*_) and the remaining for training (*𝒳*_*train*_) and validation (*𝒳*_*val*_). These 20 test set images were evenly distributed across the GA range that we used, with four images for every second GW (at 18, 20, 22, 26, and 26 GWs). Furthermore, it was ensured that the training, validation, and analysis subsets were also evenly distributed across the gestational range. All testing images were manually annotated and thus had expert ground-truth labels (as described in 3.4).

Ten test images were twice manually annotated by the same observer, in order to obtain intra-observer variability.

To obtain atlas labels for our dataset *𝒴*^*atl*^, template images had to be generated for our dataset (*K* and *K*_*clust*_). These template images were created using all images in our dataset (*𝒳*_*train*_, *𝒳*_*val*_, *𝒳*_*test*_, *𝒳*_*analysis*_), as these were available from earlier work (Namburete et al., 2018a). However, the effect on the appearance of the template images of using all images versus only the one used for training is very small, and as such not expected to have a significant effect on the resulting atlas labels.

As the GA of the dataset used in this study ranges between 18 and 26, *n*_*a*_ for the training data was set to 9. This yielded two overlapping training sets, one with 239 images containing weak atlas labels, and one with 9 images containing expert labels. During training of our networks, 10% of the 239 was used as validation. For the 9 expertly labelled individual images, no validation set was used, but all settings were kept the same as when training with atlas labels. An overview of how the data were separated into different subsets, and the number of available labels in each set is summarized in Table 2.

#### 3.5.2 Experiments

In order to study the effect of using only a small number of manual annotations, multiple experiments were performed. Models trained with either expert or atlas labels are denoted by *θ^atl^* and *θ^exp^*.

*Comparison between atlas and expert labels* Firstly, we aimed to study the effect of using *n*_*a*_ expertly annotated images versus many weakly labelled images, obtained from annotating *n*_*a*_ template images. This first experiment was performed with images from the full GA range in our data, resulting in *n*_*a*_ = 9. Defining *n*_*x*_ as the number of training images used during training, two separate networks were trained: one that used all atlas-labelled images (*θ^atl^*, *n^x^* = 215) and a second that only used the expertly labelled images (*θ^exp^*, *n*_*x*_ = 9). As the alignment of the images was expected to influence the performance, this experiment was repeated for the aligned images, $\dot{X}$, as well as for images in their original orientation, $\overset{˘}{X}$. Models trained with aligned images were evaluated on aligned testing data, and vice-versa.

##### Varying the number of manual annotations

Next, *n*_*a*_ was reduced to study the performance decrease when using even fewer manual annotations for training. For *𝒴*^exp^, a simple subset of *n*_*a*_ images were used whereas for *𝒴*^*atl*^ all images obtained from *n*_*a*_ annotated templates were used. The models trained with these labels are noted as $\theta_{n_{a}}^{\exp}$ and $\theta_{n_{a}}^{atl\;}$, and in our experiments *n*_*a*_ ranged from 2 to 9. The images used for training were selected to be uniformly distributed over the whole GA range, e.g. for *n*_*a*_ = 2 the images from 20 and 24 GWs were selected. Even though these models were only trained with images from selected GWs, evaluation was performed on all testing images. As for the previous experiment, this was repeated for both $\overset{˘}{X}$ and $\dot{X}$.

##### Cluster-based atlas labels

During initial testing, we observed that using atlas labels from multiple LPVH templates, *K^clust^*, increased performance compared to using the labels from a single annotated template per GW, *K* (see Table 4). For this reason, all aforementioned experiments using atlas labels were performed with the LPVH annotations from the clustered template images. To quantify the performance increase from these improved template images, we also annotated the LPVH in *K*, and trained a network with these labels. As this only applies to the atlas labels, no expert training labels were used in this experiment.

#### 3.5.3 Network training

To train our networks, we used a combination of multi-class DSC (ℒ_*MD*_) and Cross-Entropy (CE) loss (ℒ_*CE*_), as defined by:

(1) $${ℒ}_{total\;}={ℒ}_{MD}+\lambda {ℒ}_{CE}$$

with *λ* as a relative weighting parameter between both loss terms. It was shown during initial experiments that the DSC loss term resulted in higher performance for individual structures due to the high unbalance between the structures and background, whereas the addition of the CE loss term ensured convergence for all structures. Based on these experiments the *λ* was set to 1, but did not have a strong effect on performance for values within the same order of magnitude.

To prevent overfitting, for each sample a combination of the following geometric augmentations was performed: horizontal flips (across the midline for aligned images), rotation (±30°), translation (±10 voxels) and scaling. The scaling range (defined by *s*_min_ and *s*_max_) was set for each GW separately and given by:

(2) $$\begin{array}{l} s_{min}(w)=max\left(\sqrt[{3}]{\frac{0.9\cdot \bar{V_{brain\;}}(18))}{\bar{V_{brain\;}}(w)}},1/1.5\right)\\ s_{max\;}(w)=min\left(\sqrt[{3}]{\frac{1.1\cdot \bar{V_{brain\;}}(26)}{\bar{V_{brain\;}}(w)}},1.5\right) \end{array}$$

with *w* the GA of the fetus in weeks and $\bar{V_{brain\;}}(w)$ the average whole brain volume at a certain GW. This ensured that scaling was only performed to brain sizes within the dataset (i.e. images at 18 GW were predominantly up-scaled whereas images at 26 GW were mostly down-scaled). We applied augmentation for training with both $\dot{X}$ and $\overset{⌣}{X}$. For $\dot{X}$, this meant that the network was trained with images slightly deviating from the initial alignment to the same coordinate system, making it more robust to imperfect alignment.

As the size of the training set varies strongly across experiments, all our models were trained for the same number of iterations (defined as passing a single batch of data through the network). This number was empirically set to be equivalent to training with all atlas-labelled images for 100 epochs. We chose the ADAM optimizer (Kingma and Ba, 2014) with an initial learning rate of 0.001 for training, and used a batch size of four. This relatively small batch size was constrained by the GPU memory due to the large input images (160 × 160 × 160). The results reported in this study are the performance after the last training epoch averaged over three training runs.

#### 3.5.4 Implementation

All models were implemented in Python 3.7 using PyTorch (version 1.7.1). We trained our models on an NVIDIA Tesla V100 with 32 GB of memory. To generate the template images used to obtain our atlas labels we used Matlab (version 9.8), and all manual annotation was performed using the freely available MITK-Workbench.

### 3.6 Evaluation

#### 3.6.1 Post-processing

During visual inspection of the resulting segmentations, we noticed that for some images (about 25% of the test images for $\dot{X}$ and 50% for $\overset{⌣}{X}$), *θ*^exp^ made predictions that were also in the hemisphere proximal to the transducer (which is partly occluded due to shadowing), whereas our ground-truth only contained manual annotations of the distal hemisphere for both the CP and LPVH. However, these predictions were always smaller in size than the predictions in the distal hemisphere, as only part of the structure was clearly visible. Furthermore, for $\overset{⌣}{X}$ some very small spurious areas were predicted for a few test images. Quantitatively, this can also be seen from the resulting large Hausdorff distances before post-processing (see Appendix B, Fig. B.14). For these reasons, we post-processed our predictions Ŷ_*c*_ by taking the largest connected component as the final prediction, denoted by Ŷ_*c*,*post*_. As the evaluation metrics are computed both with and without post-processing, for simplicity the notation Ŷ_*c*_ is used in the remainder of this section to represent either case.

#### 3.6.2 Metrics

The binary predictions for each class, Ŷ_*c*_, were separately evaluated against the binary manual expert labels for the respective class, $Y_{c}^{\exp}$. For evaluation we used the Dice Similarity Coefficient (DSC) and the 95th percentile Hausdorff distance (*H*_95_). The 95th percentile Hausdorff distance was used as it is more robust to small outliers than the standard maximum Hausdorff distance. A more detailed description of the evaluation metrics can be found in Appendix E.

In addition to the DSC and *H*_95_ we also computed the signed and unsigned relative volume differences. These results are presented in Appendix B (Figs. B.3 and B.4).

### 3.7 Volumetric subcortical growth trajectories

In order to generate growth curves for the subcortical structures, we finally applied our models to the US images in our analysis subset. We predicted subcortical volumes $V_{{\hat{Y}}_{c,\;post\;}}$ for ${\dot{X}}_{analysis\;}$ using both ${\dot{\theta }}^{\exp}$ and ${\dot{\theta }}^{atl\;}$ in order to analyze the differences. Subsequently, a linear or quadratic polynomial was fit to the predicted volumes as function of the GA. The quadratic term was only added if it proved to be statistically significant for both ${\dot{\theta }}^{\exp}$ and ${\dot{\theta }}^{atl\;}$ (determined by a two-sided *t*-test), as the underlying structural growth should be the same between the two networks.

In addition to structural volumes, we also computed the relative structural volume with respect to the whole brain volume, defined by:

(3) $$V_{rel\_brain\;}=\frac{V_{{\hat{Y}}_{c,\;post\;}}}{V_{brain\;}}$$

with *V*_*brain*_ the whole brain volume of the respective image. These whole brain volumes were computed from whole brain masks derived from an MRI fetal brain atlas (Gholipour et al., 2017). The template images in this atlas, ranging from 21 to 31 GWs, where first binarized to obtain whole brain atlas masks and subsequently aligned to the individual US images using an affine transform (rigid + scale). For each US image, the template of the corresponding GW was used, and for fetuses younger than 21 GWs, the template image of 21 GWs was used.

### 3.8 Ethics statement

The INTERGROWTH-21st Project was approved by the Oxfordshire Research Ethics Committee “C” (ref: 08/H0606/139), the research ethics committee of the individual participating institutions and the corresponding regional health authorities in which the project was implemented. Participants provided written consent to be involved in the project (Villar et al., 2013).

## 4 Results and discussion

All performance values presented in this section were obtained from *Ŷ_c,post_* (predictions post-processed to only keep the largest connected component). Results without post-processing are given in Appendix B (Figs. B4 and B5).

### 4.1 Weak atlas labels

In order to quantify the amount of inaccuracies in our weak atlas training labels, we determined the DSC overlap between the naïve atlas propagated labels (*𝒴*^*atl*^) and the manual ground-truth labels (*𝒴*^exp^) in our test set. These results are shown in Table 3 as *prop. atlas*. For the LPVH, the DSC overlap of the atlas labels obtained from the clustered template images, *K* ^*clust*^, is shown. It can be observed that the naive propagated atlas labels obtained relatively low DSC, ranging from 0.68 for the LPVH to 0.80 for the CB, indicating that the atlas labels used as weak ground-truth labels contain a substantial amount of label noise.

### 4.2 Comparison between atlas and expert labels

**Fig. 4:**
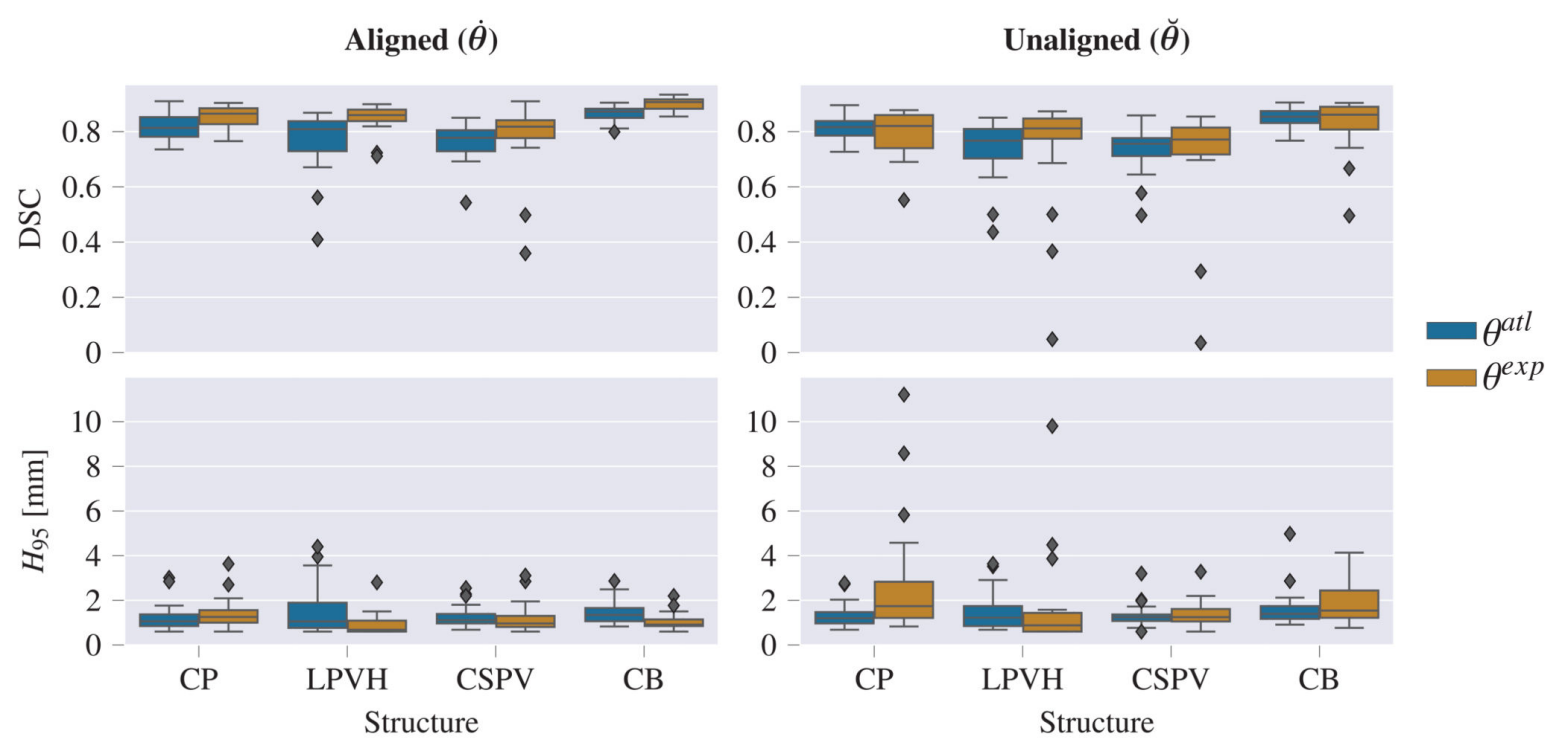
Resulting performance values after post-processing for *θ*^exp^ and *θ*^*atl*^. For the unaligned images, larger *H*_95_ are observed than for the aligned set-up, most pronounced for the *θ*^exp^. For *θ*^*atl*^, the segmentation performance between the aligned and unaligned settings shows only a small difference.

The resulting segmentation performance of our trained networks *θ*^*atl*^ (*n*_*x*_ = 239) and *θ*^exp^ (*n*_*x*_ = 9) are shown in Fig. 4 and Table 3. For both networks, the number of images that was manually annotated to obtain the training labels was the same (*n*_*a*_ = 9). The results shown were based on experiments that were trained and tested on images with the same alignment, either $\overset{˘}{X}$ or $\dot{X}$. Table 3 shows the performance for the aligned images (statistical results for these comparisons are provided in Appendix C) whereas in Fig. 4 results for both the aligned and unaligned settings are presented.

It can be observed that, somewhat surprisingly, in the aligned setting best performance is obtained using only nine expertly annotated images, as opposed to a much larger number of weakly annotated images. This indicates that even though segmentation in 3D US by a human annotator is a challenging task, a network is able to learn the correct features from only a small set of manual annotations, provided that the images are all aligned to the same coordinate space.

### 4.3 Varying the number of manual annotations

**Fig. 5:**
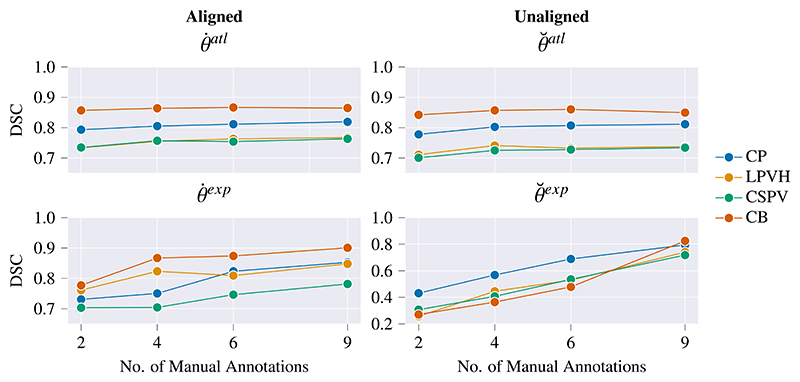
Varying the number of manual annotations (*n*_*a*_) used for generating the training labels. Reducing *n*_*a*_ has only a small effect on the performance of $\theta_{n_{a}}^{atl\;}$ whereas a drop of performance is observed for $\theta_{n_{a}}^{\exp}$. Note the different range of the y-axis of the bottom right plot.

The results of reducing the amount of manual annotations, *n*_*a*_, are shown in Fig. 5. It can be seen that for $\theta_{n_{a}}^{atl\;}$, only a slight decrease in performance is observed when decreasing *n*_*a*_. Even by training with labels generated from just 2 GWs (coming from 2 manual annotations) the performance is close to the maximum performance (average DSC of 0.78 versus maximum DSC of 0.80 for the aligned case).

On the other hand, for $\theta_{n_{a}}^{\exp}$ a performance decrease was observed when reducing the number of training images, showing a stronger decrease for the *unaligned* images than for the *aligned* images. As in this experiment we used as little as two images for training, while evaluating on images from the whole GA range, this drop in performance was expected.

### 4.4 Effect of image alignment

To explore the sensitivity of the models to brain pose, we studied the effect of image alignment on the segmentation performance in our experiments. From Fig. 4 it can be seen that higher performance was obtained for the *aligned* images compared to the *unaligned* images, which is most pronounced in the lower *H*_95_ for these images. As segmenting the structures in *aligned* images can be considered an easier task, this was in line with our expectations.

When comparing the performance between the *aligned* and *unaligned* settings for both models in more detail, a large performance difference can be observed for *θ*^exp^ between the *unaligned* and *aligned setting*, being most pronounced in Fig 5. On the other hand, this difference was not as pronounced for *θ*^*atl*^. This can be explained by the fact that in the *unaligned* setting the segmentation task consists of a combination of structure localization and subsequent segmentation. The weak, atlas labels contain imperfections at the boundaries of the segmentations, but are always approximately in the correct location. For this reason, these labels provide more information for the localization task than the few expertly annotated images and, as such, obtain better segmentation performance in the *unaligned* case. The few expertly annotated images in this setting are not sufficient to provide both localization and segmentation, especially when reducing *n*_*a*_ below nine (Fig 5). These results thus suggest that a large amount of weak labels is better for localization, whereas few expert labels perform better if the task consists of solely delineating a structure in approximately the same location.

### 4.5 Comparison to previous work

In Table 3, our resulting DSC performance using *aligned* images is compared to the intra-observer variability and to previous work performing subcortical structure segmentation. The intra-observer variability was obtained by annotating ten images from the test set twice. Furthermore, for ${\dot{\theta }}^{atl\;}$ and ${\dot{\theta }}^{\exp}$ the significance with respect to the naïve propagated atlas labels is shown. The p-values shown were obtained by a repeated measures Analysis of Variance (ANOVA) for each structure, followed by post-hoc testing with a paired *t*-test. Reported p-values are from the post-hoc testing and underwent Bonferroni correction for the four structures, as well as for the three model comparisons (see Appendix C for full statistical results).

However, it should be noted that performance metrics reported in different studies have to be carefully interpreted. Factors such as US image quality, structural definition (i.e. what is included in the segmentation), and GA range can all affect the difficulty of the task. The GA range affects the difficulty of the segmentation tasks as acoustic shadowing increases with advancing GA, due to calcification of the fetal skull. Furthermore, overlap measures, such as the DSC, generally report higher values for larger structures because a single (erroneous) voxel has a smaller effect on the resulting DSC value for these structures. We also want to stress that small performance increases are very difficult to quantify in 3D US segmentation due to the high intra- and inter-observer variability predominantly resulting from the ambiguity of the exact location of structural boundaries.

For the CP, LPVH, and CB, superior performance is obtained compared to previous work. The best performance obtained for these structures is also very close to the intra-observer variability in our test data (DSC of 0.85, 0.85 and 0.90 versus an intra-observer variability of 0.86, 0.85 and 0.91 for the CP, LPVH and CB, respectively). An especially large performance increase was observed for the CB (DSC of 0.90 versus DSC of 0.80, 0.63 and 0.73 in previous studies). However, part of this increase can be attributed to the fact that in this study the CB was manually annotated including the bright echogenic boundaries, thus resulting in a larger segmented region, whereas this boundary was not included in (Venturini et al., 2020 and Yaqub et al., 2013). This choice was made based on the fact that the transverse cerebellar diameter, which is clinically used to assess growth during a standard fetal examination, also includes this boundary (Prayer et al., 2017). Furthermore, manual segmentation is more consistent when including this boundary as the outside edge is generally clearly visible in the US images.

For the CSPV, slightly higher performance (DSC of 0.81 versus 0.78) was reported in (Huang et al., 2018), but was obtained in the 2D mid-sagittal plane. Since the lateral boundaries of the CSPV are hardest to define in 3D images, this naturally results in larger prediction errors than 2D segmentations in this plane. In contrast to the other segmented structures, the CSPV segmentation performance is also substantially lower than the intra-observer variability (DSC of 0.78 versus 0.86). This can, however, be largely attributed to poor segmentation performance at 18 GWs, when the CSPV is small and the boundaries are ill-defined (Fig. 7), as well as to the aforementioned difficulty to consistently define the lateral boundaries of the CSPV.

Due to the differences in structural definition (Venturini et al., 2020; Yaqub et al., 2013), differences in study design (i.e. 2D segmentation (Huang et al., 2018) or a limited search region (Yaqub et al., 2013) as well as unavailability of code, it was not possible to do a direct evaluation of past methods on our dataset. Although the reported comparisons should thus be interpreted with care, they do show that we obtained competitive segmentation performance.

### 4.6 Qualitative results

**Fig. 6:**
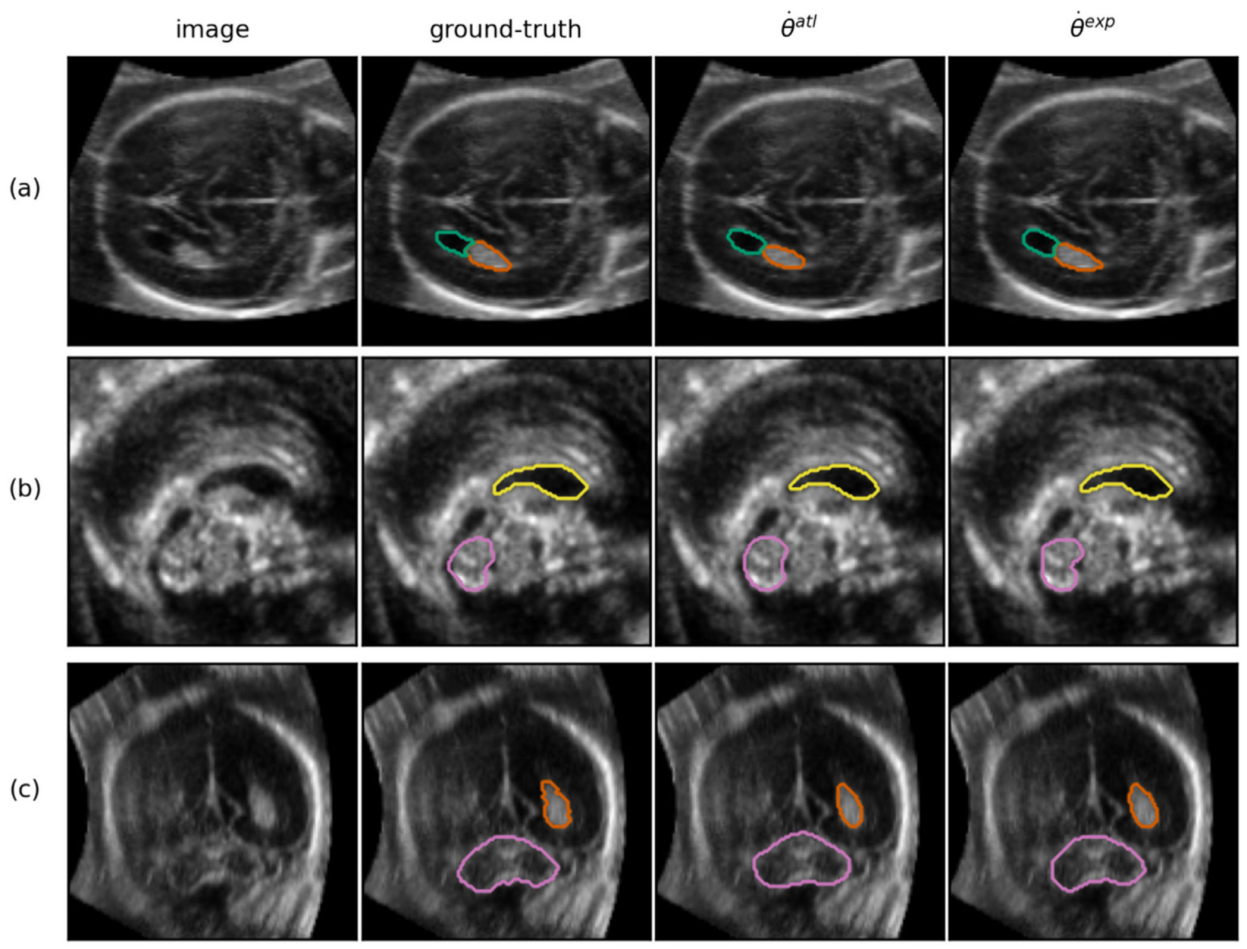
Example prediction of a test set volume at 22 GW (randomly chosen from 5 best performing images, average DSC across structures of 0.85), visualized in the axial (a), sagittal (b) and coronal (c) plane (segmentation was performed in 3D). The structures shown are the LPVH (green), CP (orange), CSPV (yellow), and the CB (pink). Only the outer boundary of the segmentation is shown in color for visualization purposes.

**Fig. 7:**
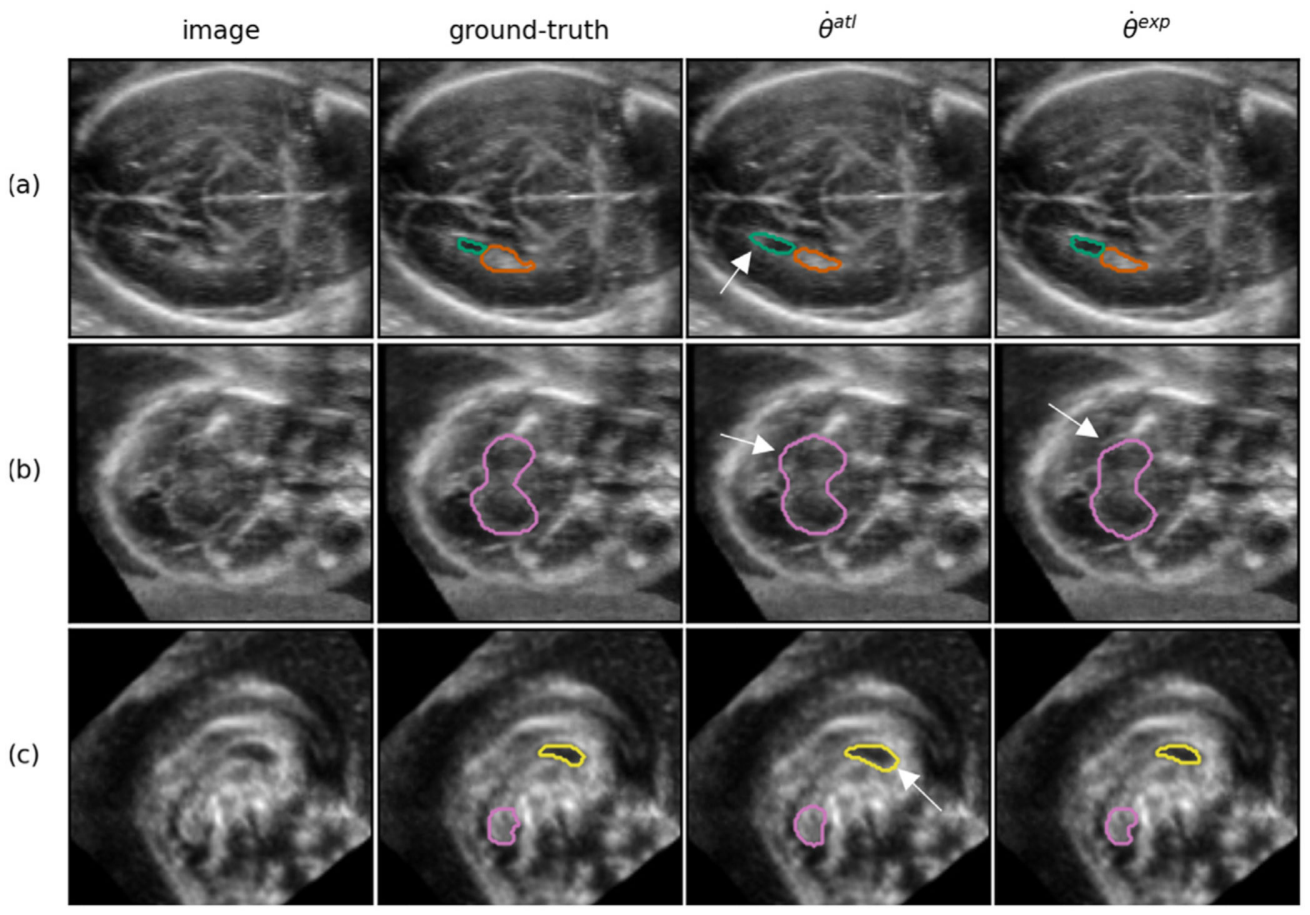
A set of examples showing the most prominent segmentation errors from ${\dot{\theta }}^{\exp}$ and ${\dot{\theta }}^{atl\;}$ after post-processing (a) axial plane of an image from a subject at 26 GW demonstrating over-segmentation of the LPVH (green) (b) axial plane of an image from a subject at 22 GW demonstrating over-segmentation of the CB (pink) for ${\dot{\theta }}^{atl\;}$ and slight under-segmentation for ${\dot{\theta }}^{\exp}$ (c) sagittal plane of an image from a subject at 18 GW, demonstrating over-segmentation for ${\dot{\theta }}^{atl\;}$ as well as the difficulty of manually segmenting the CSPV (yellow) at young ages.

In Fig. 6, an example prediction is shown for an aligned image at 22 GWs. The manual-ground truth as well as the predictions from both ${\dot{\theta }}^{\exp}$ and ${\dot{\theta }}^{atl\;}$ are shown. A qualitative inspection suggests that the predictions of the two networks closely resemble each other as well as the ground-truth. In Fig. 7, slices are shown containing the most prominent error modes in the aligned test set. From Fig. 7a-c, it can be observed that ${\dot{\theta }}^{atl\;}$ tends to over-segment in some areas whereas the predictions from ${\dot{\theta }}^{\exp}$ align better with the ground-truth. In Fig. 7c a CSPV segmentation at 18 GW with a very low DSC (0.51) for ${\dot{\theta }}^{atl\;}$ is shown. It can be observed from this figure that the CSPV is challenging to (manually) segment at this young GA due to the small structural volumes as well as to the boundaries that are not yet very well-defined.

### 4.7 Clustered LPVH labels

We extended our atlas-based labels with a clustering approach for the LPVH. This approach was only applied for the LPVH, as the other structures did not display as much shape variation within GWs. In Table 4 the LPVH segmentation performance of training ${\dot{\theta }}^{atl\;}$ with atlas labels either propagated from *𝒦* or *𝒦*^*clust*^ is shown. Training a network with the propagated clustered template annotations significantly improved the DSC performance from 0.69 to 0.77 (p<0.001) and *H*_95_ from 2.2 to 1.6 (p<0.05). Significance values were calculated using a paired *t*-test. This thus suggests that the additional variation captured by the clustered templates contributed to an improved segmentation of the LPVH.

### 4.8 Subcortical growth curves

**Fig. 8:**
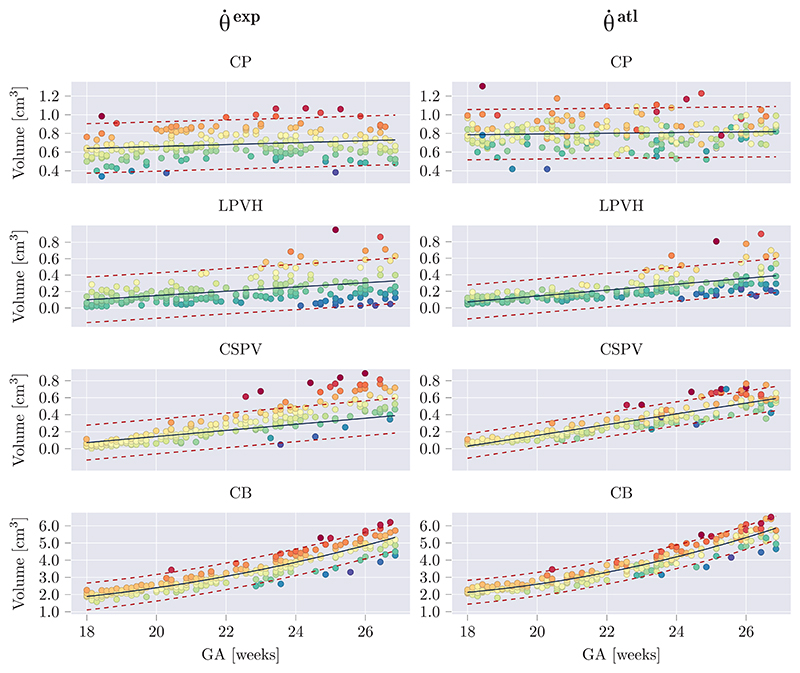
Estimated structural volumes for subcortical structures as a function of GA. Volumes were fitted with a linear or quadratic fit (black), in which the quadratic term was only added if it was significant for both networks (per structure). The 95% prediction confidence intervals where also computed and are shown with red dashed lines. For each structure, samples were colored based on their residual for ${\dot{\theta }}^{\exp}$, and the same colors (per sample) where used for ${\dot{\theta }}^{atl\;}$.

**Fig. 9:**
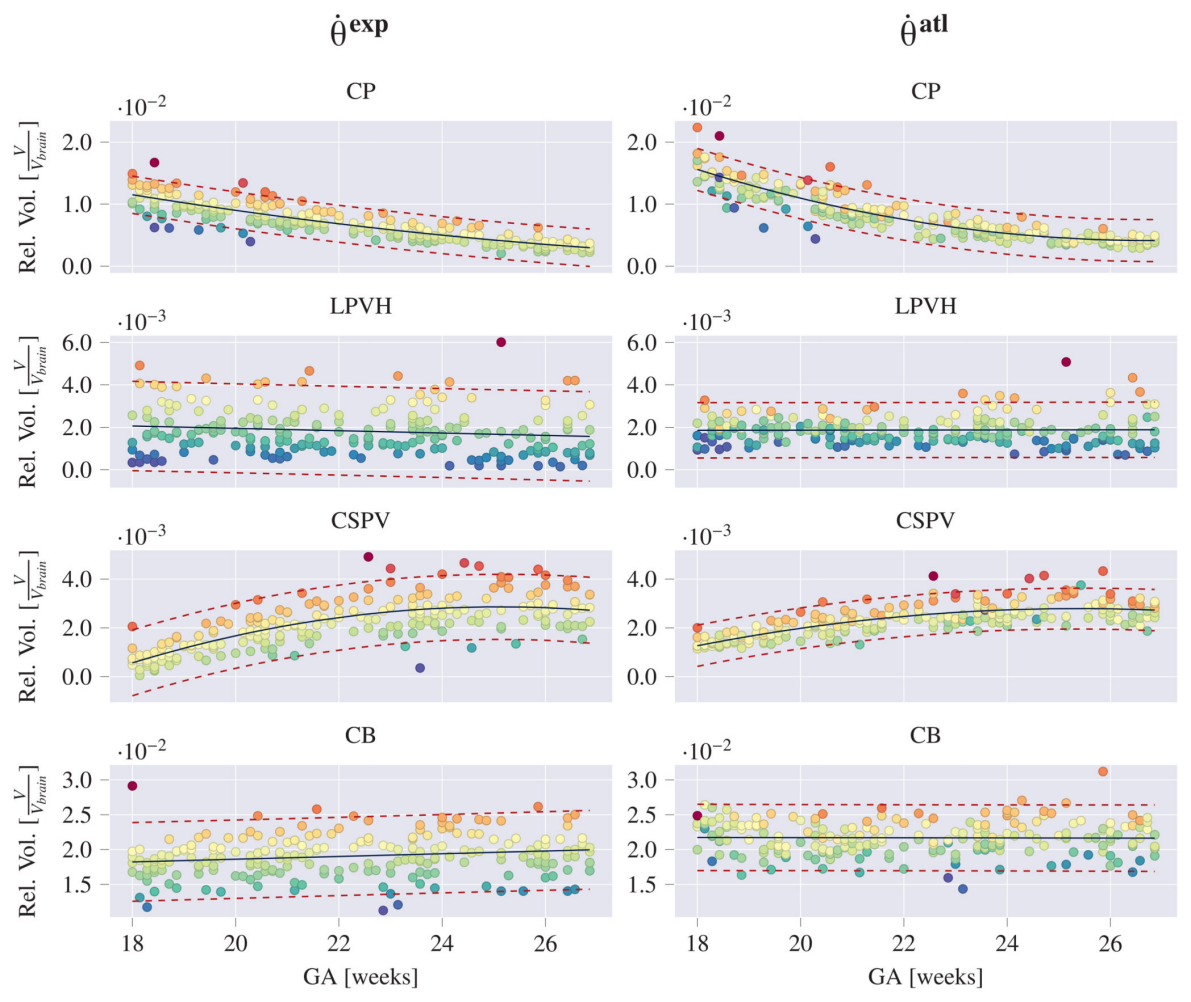
Estimated relative volumes (Rel. Vol.) with respect to the whole brain volumes (*V*_*rel*_*brain*_) for subcortical structures as a function of GA. Volumes were fitted with a linear or quadratic fit (black), in which the quadratic term was only added if it was significant for both models (per structure). The 95% prediction confidence intervals where also computed and are shown with red dashed lines. For each structure, samples were colored based on their residual for ${\dot{\theta }}^{\exp}$, and the same colors (per sample) where used for ${\dot{\theta }}^{atl\;}$.

The growth curves obtained from applying our trained networks to 278 images of healthy fetuses are presented in Figs. 8 and 9. In Fig. 8 the predicted structural volumes are shown, whereas in Fig. 9 the predicted relative volumes with respect to the whole brain volume (*V*_*rel*_*brain*_), are shown. The growth curves were fitted with a linear or quadratic fit based on the significance of the quadratic component computed with a two-sided *t*-test. All parameters obtained through fitting are given in Appendix D.

To illustrate the level of consistency between ${\dot{\theta }}^{\exp}$ and ${\dot{\theta }}^{atl\;}$, in Figs. 8 and 9 the samples were colored based on their residual value in the growth curves obtained from ${\dot{\theta }}^{\exp}$. For a certain structure, samples with matching colors between the growth curves of the ${\dot{\theta }}^{\exp}$ and ${\dot{\theta }}^{atl\;}$ thus correspond to the same image. The choice of coloring the samples based on ${\dot{\theta }}^{\exp}$ was made because of its better DSC performance (Table 3), but as it is only used to visualize the matching images, this choice is relatively arbitrary.

It can be observed that both of the models, ${\dot{\theta }}^{\exp}$ and ${\dot{\theta }}^{atl\;}$, result in a similar growth trend and that the relative sample distribution is largely preserved (i.e. values above or below the growth curve are respectively above or below the curve for the other method, by a similar amount). It can also be seen that ${\dot{\theta }}^{atl\;}$ predicts larger volumes than ${\dot{\theta }}^{\exp}$, which was also confirmed by the fact that ${\dot{\theta }}^{atl\;}$ resulted in positive unsigned relative volume differences, corresponding to over-segmentation (see Appendix B Fig. B.3). However, as the visualization of structural boundaries can be subjective in US, a consistent (albeit overestimating) segmentation demonstrates the utility of this method in comparison to manual delineation.

Previous work on volumetric subcortical development during gestation from US has largely been limited to cerebellar and thalamic growth (Babucci et al., 2019; Benavides-Serralde et al., 2009; Sotiriadis et al., 2012; Zeng et al., 2015). Therefore, to the best of our knowledge, this study provides novel US-specific volumetric growth curves of the CSPV, CP and LPVH for a geographically diverse, healthy population. To validate the volumetric measures obtained in this study, we compared the growth curves of the structural CB volume to previously reported CB growth curves (Andescavage et al., 2017; Babucci et al., 2019; Chang et al., 2000; Hata et al., 2007; Hatab et al., 2008; Kyriakopoulou et al., 2017; Scott et al., 2012) (see Fig. B.6). This showed that our curves showed excellent agreement with past US studies, especially in the first half of the GA range. After 24 GWs the growth rates of the CB (slope of the growth curves) found in this study are slightly lower than in previous work. However, as described previously, the CB becomes more challenging to segment with advancing GA and, as such, this could explain the slightly deviating volume measurements at the end of the second trimester.

From the resulting growth curves (Figs. 8 and 9), it can be observed that the CB undergoes rapid growth during the second trimester of gestation, whereas the relative cerebellar volume, with respect to the total brain volume, remains mostly consistent (between 1.5% and 2.5%). As the transverse cerebellar diameter is expected to increase linearly during the second trimester (Malinger et al., 2020), this matches the quadratic growth of CB volume. The CP only shows a very small increase in structural volumes during the second trimester. However, the relative volumes show a rapid decline with respect to the total brain volume, ranging from 12% of the total brain volume at 18 GWs to 3% at 27 GWs. This aligns with the observation that early in the second trimester the CP almost completely fills the lateral ventricles and comprises a large part of the fetal brain, whereas later in gestation it appears as a small structure only partly filling the lateral ventricles. The LPVH structural volume increases linearly from about 0.1 cm^3^ to 0.3 cm^3^ in the studied GA range, which is in agreement with the fact that the atrial width remains stable in healthy fetuses during the second trimester (Malinger et al., 2020). The CSPV shows a linear increase in structural volume during the studied GA range (from about 0.01 cm^3^ at 18 GWs to 0.6 cm^3^ at 26 GWs) but its relative volume increases early in the second trimester and remains constant after approximately 24 GWs. As a deviating measurement of the CSPV has been related to agenesis of the corpus callosum (Karl et al., 2017) but is not widely studied, the presented growth curves might facilitate this in future work.

### 4.9 Limitations and further work

In this study, growth curves were generated using networks trained on only a handful of manual annotations. Even though the segmentation performance was shown to be close to intra-observer variability, more accurate model predictions might be obtained using additional annotations for training. Furthermore, due to the high variability in manual annotations, a more accurate ground-truth could be achieved by consensus segmentations of multiple annotators.

Another limitation of this study is that no methods were used that were specifically tailored for training with noisy labels (the *atlas* labels), or for training with only a handful of annotations (the *expert labels*). However, due to the high intra-observer variability as well as the relatively small test set, it is challenging to measure small increases in segmentation performance potentially obtained by these methods. Additionally, we believe that our experiments demonstrate the feasibility of subcortical segmentation in fetal US by showing that good performance (i.e. close to human-level performance) can be obtained using simple methods that can easily be reproduced.

We have validated our methods by generating subcortical growth curves in a healthy population from US images acquired with the same US device. In further work the models developed in this study offer the promise of comparing subcortical development between different fetal cohorts without doing time-consuming manual annotations. However, more work is necessary to extensively validate the segmentation performance for datasets acquired with different US scanners as well as for cohorts containing a range of pathological conditions.

## 5 Conclusion

In summary, we showed that only a small number of annotated images are needed to successfully train a network for subcortical segmentation in 3D US images. To obtain optimal performance, alignment of the images is required beforehand, however, on unaligned testing images, a high performance can still be achieved using several weakly annotated images. By applying our trained networks to a large cohort of fetuses, we were able to generate novel US-specific growth trajectories of the CP, LPVH, CSPV and CB for a geographically diverse, healthy population. This study thus demonstrates the feasibility of subcortical segmentation in 3D US using deep learning, and shows that volumetric measures obtained from these models can be used to obtain an improved understanding of subcortical growth during gestation.

## Supplementary Material

Appendix

## Figures and Tables

**Fig. 1:**
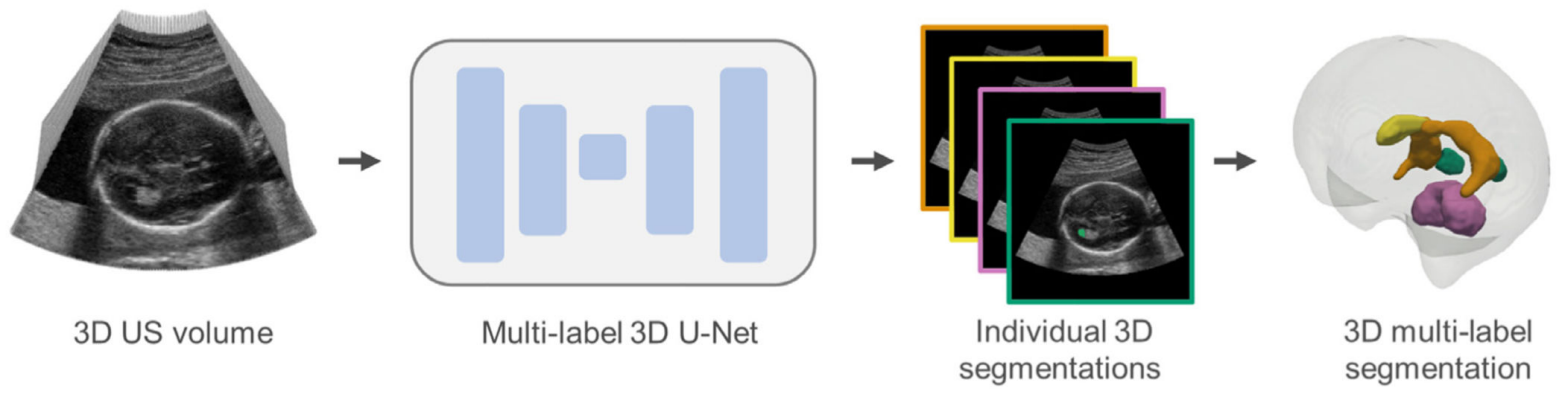
Schematic overview of segmentation network. The segmented structures shown are the CB (pink), LPVH (green), CSPV (yellow) and CP (orange). The LPVH and CP are shown in both hemispheres for visualization purposes, but are only segmented in the visible hemisphere. A more detailed Figure of the U-Net architecture can be found in Fig. B.2 (Appendix B).

**Table 1:** Overview of manual segmentation protocol. The numbers in the right top corner of each example image indicate the plane that is shown (corresonding to the planes annotated in the 3D brain visualization).

Structure	Segmentation Protocol	Example
CP (*Choroid Plexus)* 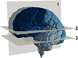	Identify in transventricular plane as circular echogenic structure inside ventricular cavity. Segment complete CP, which moves from the midline (posteriorly) to a more lateral location (inferior). Use all three planes, predominantly the axial and coronal, to ensure consistent segmentation.	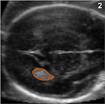
LPVH *(Lateral Posterior Ventricle Horn)*	Ventricular cavity posterior to the CP. Identify in transventricular plane as hypoechoic (dark) cavity. Segment inside echogenic boundary. Use all three planes to ensure consistent segmentation.	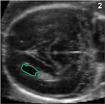
CSPV (*Cavum Septum Pellucidum et Vergae*)	Identify in midsagittal plane as fluid-filled cavity. In older fetuses the cavum septum pellucidum (anterior) and cavum vergae (posterior) can be seen as two adjoining cavities but are segmented together. Use axial plane to determine lateral boundaries of CSPV.	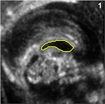
CB (*Cerebellum)*	Include bright echogenic boundary^a^. Identify on axial plane as the two cerebellar hemispheres connected by the vermis. Determine superior and inferior boundaries in sagittal plane. Use all three planes to ensure consistent segmentation.	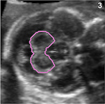

a Cerebellar boundary included for consistency as transverse cerebellar diameter is clinically measured including this boundary

**Table 2:** Overview of the number of images and available labels for each of the data subsets.

	volumes	atlas labels	expert labels
Analysis subset	278	0	0
Model Development subset			
Testing	20	20	20
Training	215	215	9
Validation	24	24	0

**Table 3:** DSC performance of our segmentation networks using aligned images compared to results of previous studies (obtained on different datasets). Numbers in between brackets indicate the standard deviation and the best performance for each structure is shown in bold. For both ${\dot{\theta }}^{\exp}$ and ${\dot{\theta }}^{atl\;}$ results of statistical testing with respect to the propagated atlas labels are shown with (**) *p* <.005 and (*) *p* <.05. Full overview of statistical results can be found in Appendix C.

	CP	LPVH	CSPV	CB
Gutiérrez-Becker et al. (2013)				0.80 (0.05)
Yaqub et al. (2013) ^a^	0.79 (0.09)	0.82 (0.10)	0.74 (0.11)	0.63 (0.15)
Huang et al. (2018) ^b^	0.76 (0.08)		**0.81** (0.06)	
Venturini et al. (2020)				0.73 (0.01)
This work				
prop. atlas	0.79 (0.07)	0.68 (0.10)	0.72 (0.10)	0.80 (0.09)
${\dot{\theta }}^{atl\;}$	0.82 (0.05)**	0.77 (0.11) ^**^	0.76 (0.07)	0.86 (0.03)*
${\dot{\theta }}^{\exp}$	**0.85** (0.04)**	0.85^**^ (0.05)	0.78 (0.13)	**0.90** (0.02)**
Intra-observer variability	0.85 (0.05)	0.86 (0.03)	0.86 (0.04)	0.91 (0.03)

a Search region limited to cuboid around ground-truth annotation

b Segmentation in 2D standard planes

**Table 4:** LPVH segmentation performance using two different types of atlas-based annotation: LPVH labels obtained from annotating a single template per week (*𝒦*) or annotating a set of clustered template images (*𝒦*^*clust*^).

Network (template type)	DSC	*H_95_* [mm]
${\dot{\theta }}^{atl\;}(𝒦)$	0.69 (0.13)	2.2 (1.40)
${\dot{\theta }}^{atl\;}\left({𝒦}^{clust\;}\right)$	0.77 (0.11)	1.6 (1.2)

## Data Availability

The image data is available from the INTERGROWTH-21st Consortium upon reasonable request. All network training code has been made available on: https://github.com/lindehesse/FetalSubcortSegm_Code, and weights of the trained networks can be requested by emailing the corresponding author. The groupwise registration algorithm used to generate the template image in this work was adopted from (Namburete et al., 2018a), and requests for this code can be addressed to the corresponding author of that paper.
