# Supplementary material for: Subcortical segmentation of the fetal brain in 3D ultrasound using deep learning
Source: Neuroimage. Author manuscript; Available in PMC 2026 Mar 14. (PMC7618871; doi:10.1016/j.neuroimage.2022.119117)
Supplement: Appendix [file EMS212828-supplement-Appendix.pdf]

Appendix A

List of Notations

Table A1
List of notations used in this work.

Symbol	Description
X	3D US image
$\dot{X}$	3D US image, rigidly aligned to the same coordinate system
$\breve{X}$	3D US image in the original acquired orientation
Y	multi-label segmentation mask
$\hat{Y}$	Predicted multi-label segmentation mask
Y_c, $\hat{Y}_c$	Binary segmentation mask of a single class c
Y^{type}	Atlas ($type = atl$) or expert ($type = exp$) labels
$\hat{Y}_{c,post}$	Predicted multi-label segmentation mask of a single class, post-processed with largest connected component
θ^{type}	Network trained with expert or atlas labels
$\dot{\theta}^{type}$, $\breve{\theta}^{type}$	Networks trained with $\dot{X}$ and $\breve{X}$, respectively
n_a	Number of annotated (template) images
n_x	Number of images used for training
n_v	Number of voxels per image
n_c	Number of clusters used for cluster-based template construction
$\theta^{type}_{n_a}$	Network trained with labels from n_a manually annotated (template) images
θ^{type}_{2GW}	Refers to networks that were trained per two-week gestational window
T	Non-rigid transformation to transform an image to a reference space
K	(Annotated) template image created using the standard template construction
K^{clust}	(Annotated) template image created using a clustering approach
$\mathcal{X}, \mathcal{Y}, \mathcal{K}, \mathcal{T}$	Sets of X, Y, K and T, respectively, following all conventions as described above
$\mathcal{S}$	Subset of $\mathcal{X}$
$\overline{V_{brain}}(w)$	Average whole brain volume (in the dataset used) at gestational week w
V_{brain}	Whole brain volume of an individual image
B_c, $\hat{B}_c$	Surface points of Y_c and $\hat{Y}_c$, respectively.
ΔV_{rel}	Signed relative volume difference between ground-truth and network prediction (with respect to the ground-truth structure size)
$\|\Delta V_{rel}\|$	Unsigned relative volume difference between ground-truth and network prediction (with respect to the ground-truth structure size)
V_{rel_brain}	Relative structural volume (with respect to the whole brain volume)

Appendix B

Additional Figures

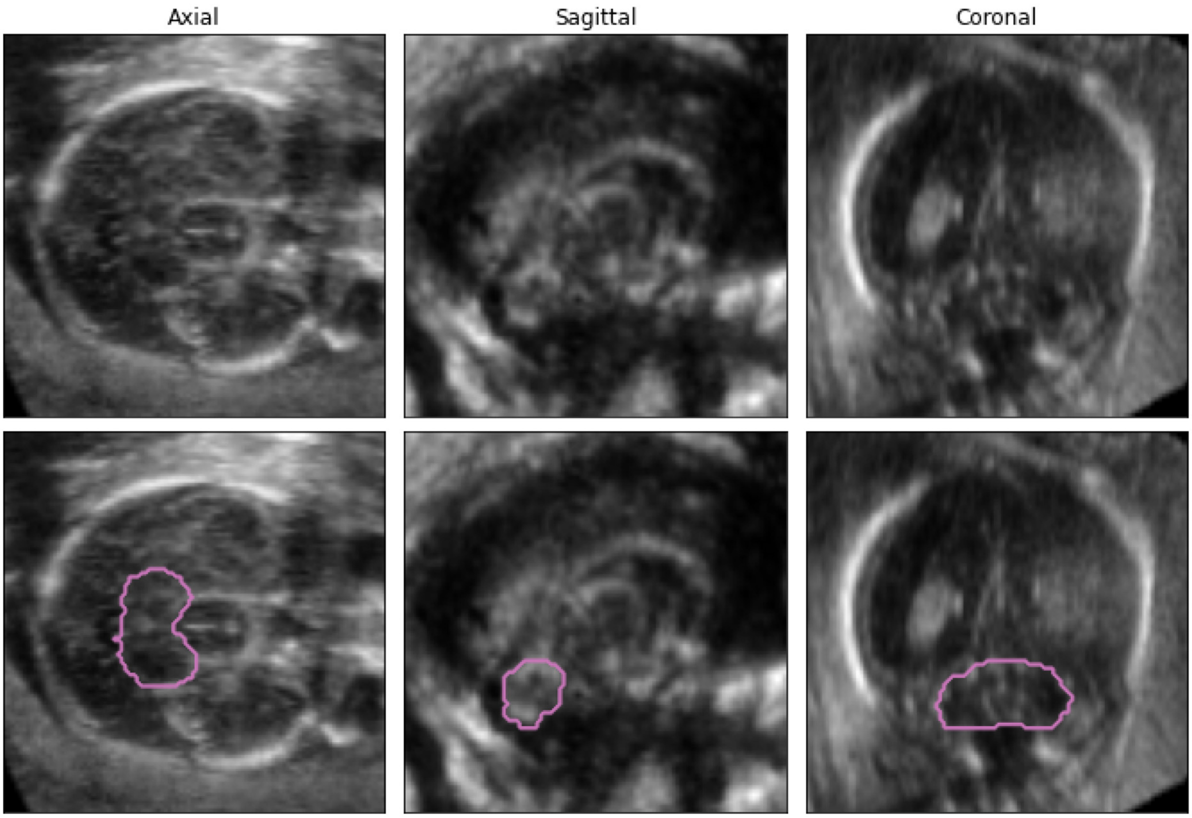

Fig. B1. Example of a challenging manual annotation for the CB. The three columns each indicate one of the orthogonal 2D views. The top row shows the image without the annotation, and in the bottom row the outer boundaries of the manual labels are shown in pink on top of the image. It can be observed that for this image in both the axial and coronal plane the cerebellar boundaries are very hard to distinguish, whereas on the sagittal view the bean shaped CB is better visible.

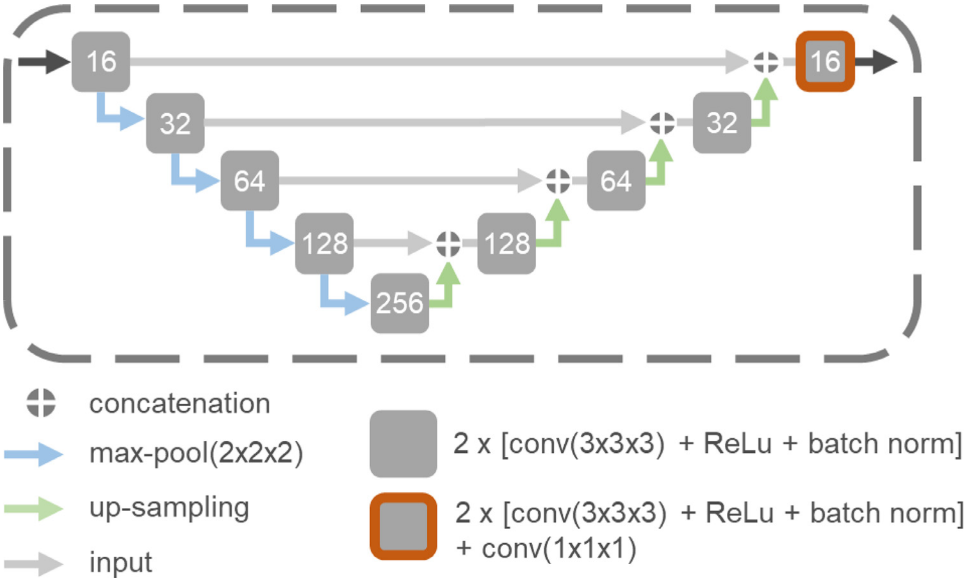

Fig. B2. Overview of the 3D U-Net architecture used in this study. The numbers on top of the grey blocks indicate the number of feature maps in the respective layer. The input is a 3D ultrasound volume of size 160x160x160 and the network outputs a multi-class segmentation of the same dimensions.

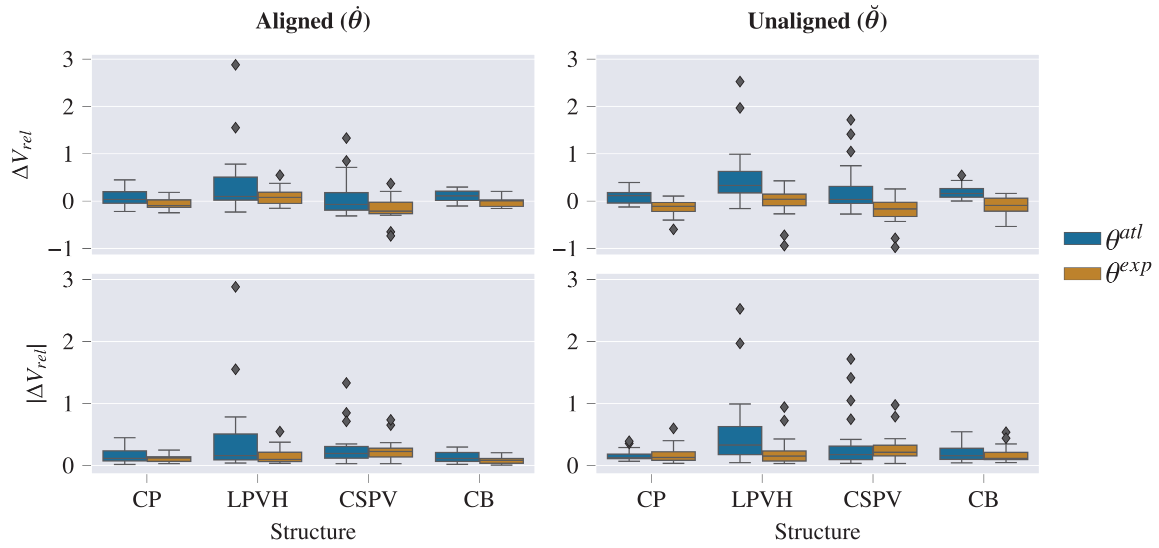

Fig. B3. Signed and unsigned relative volume differences after post-processing for $\theta^{\exp}$ and θ^{atl} after largest component post-processing. θ^{atl} shows an average ΔV_{rel} higher than 0 (except for the CSPV in the aligned setting), corresponding to over-segmentation. $\theta^{\exp}$ shows, on average, a slight negative ΔV_{rel}, thus under-segmentation, for the unaligned images while the differences are centered around zero for the aligned data. The clear outliers for the CSPV and LPVH correspond to images at 18 or 20 GW where the total ground-truth volume of the respective structures is very small, resulting in large relative volume differences.

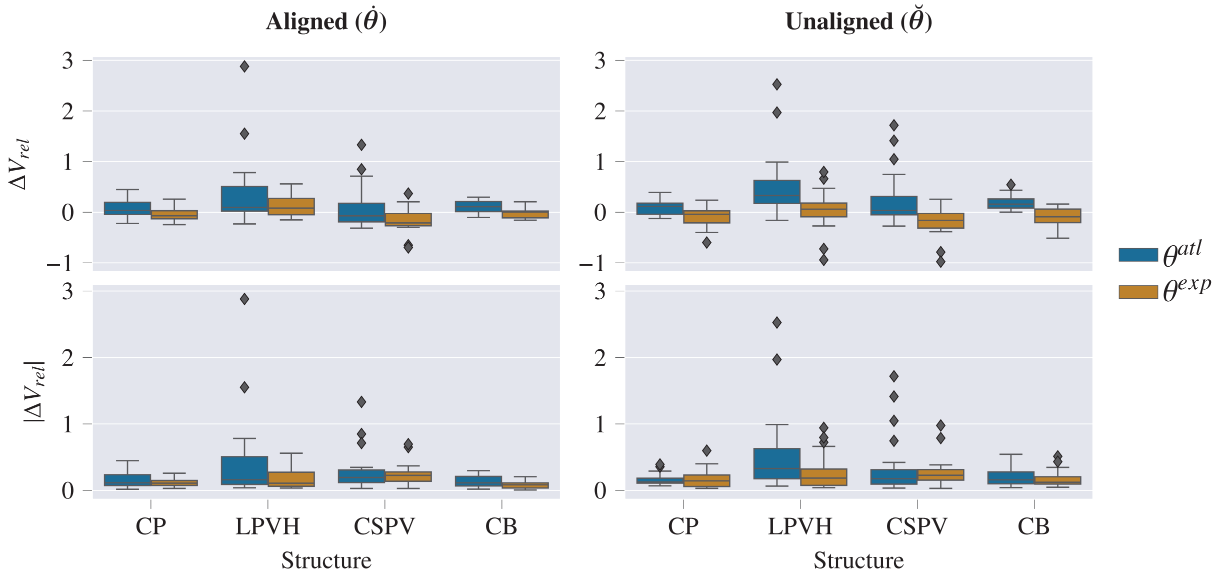

Fig. B4. Signed and unsigned relative volume differences before the largest connected component post-processing step was applied for both $\theta^{\exp}$ and θ^{atl}. It can be seen that post-processing has only a very small effect on the resulting volume measurements (i.e. compared to Fig B.3).

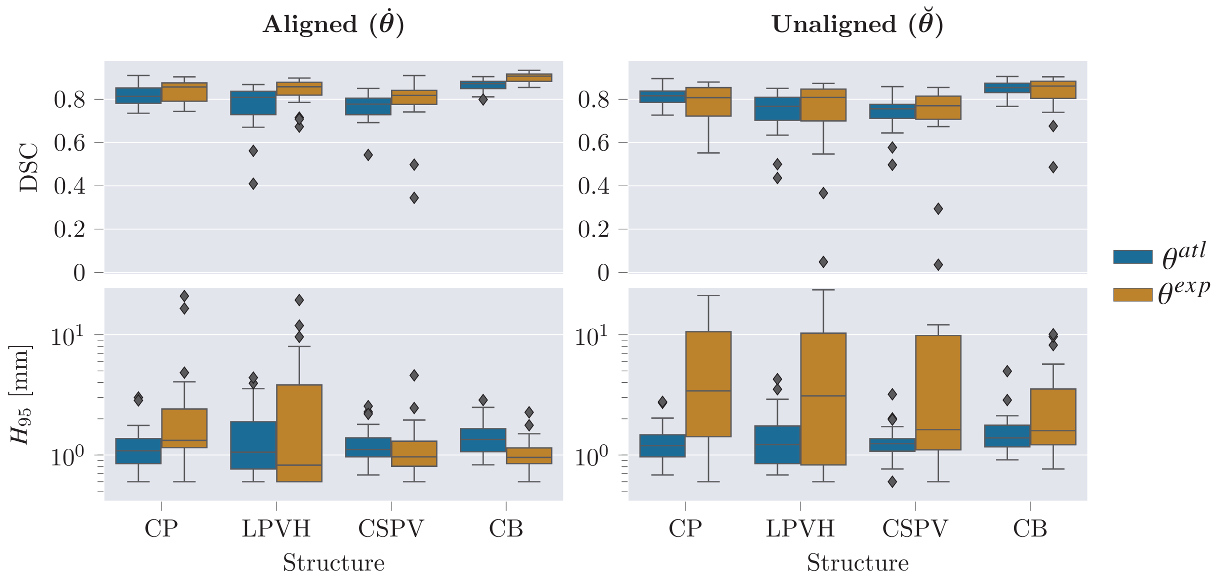

Fig. B5. Resulting performance values before the largest connected component post-processing step was applied for θ^{exp} and θ^{atl}. Note the log scale for the y-axis of the bottom row. It can be seen that the reported Hausdorff distances are without post-processing are large, which is due to spurious segmented regions, predominantly in the less-visible hemisphere.

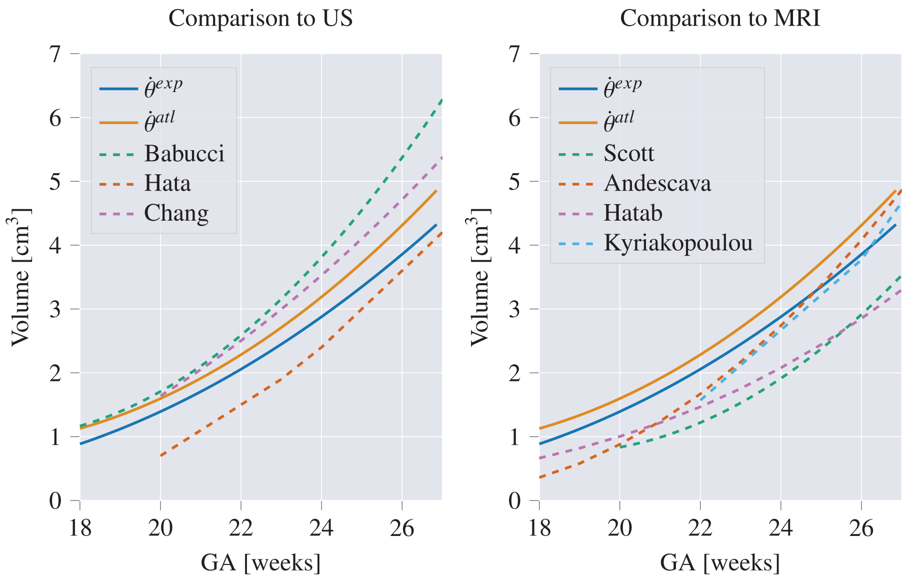

Fig. B6. Comparison of growth curves obtained for the CB in this study to previous work. In the left panel the CB growth curves from our study ($\dot{\theta}^{\text{exp}}$ (blue) and $\dot{\theta}^{atl}$ (orange)) are compared to other studies reporting cerebellar growth curves from US: Babucci Babucci et al. (2019) (green), Hata Hata et al. (2007) (dark orange), Chang Chang et al. (2000) (pink), and the right panel compares our curves to previously reported MRI growth curves: Scott Scott et al. (2012) (green), Andescava Andescavage et al. (2017) (dark orange), Hatab Hatab et al. (2008) (pink), and Kyriakopoulou Kyriakopoulou et al. (2017) (light blue).

Appendix C

Statistical Testing

Table C1
P-values obtained from comparing the DSC segmentation performance of both networks, $\hat{\theta}^{atl}$ and $\hat{\theta}^{\exp}$, with each other as well as with the naive propagated atlas masks (*prop. atlas*). For each structure a repeated measures ANOVA was performed followed by post-hoc testing with a paired *t*-test. Reported p-values are post-hoc tests corrected with Bonferonni correction for the four structures, as well as for the three model comparisons. For the CSPV the ANOVA returned non-significant differences (p=0.08), and as such the post-hoc tests are not reported.

	prop. atlas vs $\hat{\theta}^{atl}$	prop. atlas vs $\hat{\theta}^{\exp}$	$\hat{\theta}^{atl}$ vs $\hat{\theta}^{\exp}$
CP	<0.005	<0.001	<0.001
LPVH	<0.001	<0.001	<0.01
CSPV	-	-	-
CB	<0.05	<0.005	<0.001

Appendix D

Growth Trajectories

Table D1
Parameters resulting from the fitting of the structural volumes as function of the gestational age ($V = bias + x_1 w + x_2 w^2$, with w the age given in weeks) for the different subcortical structures.

	model	bias	x_1	x_2	significance x_2
CP	expert	0.7008	0.015	-0.0054	$p < 0.001$
	atlas	0.8204	0.0125	0.0024	$p = 0.065$
LPVH	expert	0.2072	0.0241	-0.0004	$p = 0.79$
	atlas	0.2199	0.0354	0.0016	$p = 0.085$
CSPV	expert	0.2857	0.0668	0.0009	$p = 0.38$
	atlas	0.2986	0.0610	0.0610	$p < 0.05$
CB	expert	2.3112	0.3826	0.0197	$p < 0.001$
	atlas	2.6064	0.3924	0.0222	$p < 0.001$

Table D2
Parameters resulting from the fitting of the relative volumes (with respect to the whole brain volume) as function of the gestational age ($V_{rel\_brain} = bias + x_1 w + x_2 w^2$, with w the age given in weeks) for the different subcortical structures.

	model	bias	x_1	x_2	significance x_2
CP	expert	0.071	-0.0010	$4.654\ 10^{-5}$	$p < 0.005$
	atlas	0.084	-0.0013	0.0001	$p < 0.001$
LPVH	expert	0.0018	$-7.785\ 10^{-5}$	$-5.397\ 10^{-6}$	$p = 0.6$
	atlas	0.0019	$-4.979\ 10^{-6}$	$-4.017\ 10^{-6}$	$p = 0.5$
CSPV	expert	0.0021	0.0002	$-4.697\ 10^{-5}$	$p < 0.001$
	atlas	0.0024	0.0001	$-2.916\ 10^{-5}$	$p < 0.001$
CB	expert	0.0914	0.0001	$-3.002\ 10^{-5}$	$p = 0.27$
	atlas	0.0224	-0.0003	$5.121\ 10^{-5}$	$p < 0.05$

Appendix E. Evaluation Metrics

The following evaluation metrics were used for evaluation, with $Y_c^{\exp}$ a binary manual expert label in the test set, and $\hat{Y}_c$ the predicted binary masks for a single class:

- The Dice Similarity Coefficient (DSC), defined by:

$$DSC(Y_c^{exp}, \hat{Y}_c) = \frac{2 \cdot \sum_{i=1}^{n_v} Y_{c,i}^{exp} \cdot \hat{Y}_{c,i}}{\sum_{i=1}^{n_v} Y_{c,i}^{exp} + \sum_{i=1}^{n_v} \hat{Y}_{c,i}} \tag{E.1}$$

with n_v the total number of voxels per image. A DSC of 1 indicates perfect overlap between the ground-truth and prediction, whereas a DSC of 0 indicates no overlap.

- The 95th percentile Hausdorff distance (H_{95}), which is the maximum (or 95th percentile in our case) over the set of boundary points of the minimum distances (between a boundary point in one set and any boundary point in the other set). If B_c and $\hat{B}_c$ represent the surface points of Y_c and $\hat{Y}_c$ respectively, the H_{95} is given by Huttenlocher et al. (1993):

$$H_{95}(B_c, \hat{B}_c) = \max(h_{95}(B_c, \hat{B}_c), h_{95}(\hat{B}_c, B_c)) \tag{E.2}$$

where h is the directional Hausdorff distance given by:

$$h_{95}(A, B) = 95th_{\mathbf{a} \in A} \min_{\mathbf{b} \in B} d(\mathbf{a}, \mathbf{b}) \tag{E.3}$$

with $d(\mathbf{a}, \mathbf{b})$ the Euclidean distance between points **a** and **b**, and 95th being the 95th percentile. We are using the 95th percentile distance because it is more robust to very small outliers than the standard maximum Hausdorff distance. As the Hausdorff distance represents the distance between prediction and ground-truth, lower distances indicate better segmentation performance.

- The signed (ΔV_{rel}) and unsigned ($|\Delta V_{rel}|$) relative volume differences, defined by:

$$\Delta V_{rel} = \frac{V_{\hat{Y}_c} - V_{Y_c^{exp}}}{V_{Y_c^{exp}}} = \frac{\sum_{i=1}^{n_v} \hat{Y}_{c,i} - \sum_{i=1}^{n_v} Y_{c,i}^{exp}}{\sum_{i=1}^{n_v} Y_{c,i}^{exp}} \tag{E.4}$$

$$|\Delta V_{rel}| = \frac{|V_{\hat{Y}_c} - V_{Y_c^{exp}}|}{V_{Y_c^{exp}}} \tag{E.5}$$

with V_{Y_c} the volume of Y_c. The signed difference indicates whether the model's prediction is over- or under-segmented. A positive number indicates over-segmentation, whereas a negative number indicates under-segmentation. On the other hand, the unsigned volume difference indicates the error in the volumetric measures predicted by the segmentation network. Given that the segmentation networks were developed in order to extract volumetric information of the structures, this measure is important in estimating the expected error. As the structures segmented in this study vary considerably in size, the relative volume differences (with respect to the ground-truth structural volume) enable comparison across the structures.
